# Analysis of patchclamp recordings: model-free multiscale methods and software

**DOI:** 10.1007/s00249-021-01506-8

**Published:** 2021-04-09

**Authors:** Florian Pein, Benjamin Eltzner, Axel Munk

**Affiliations:** 1 Statistical Laboratory, DPMMS, University of Cambridge, Cambridge, UK; 2 Institute for Mathematical Stochastics, Georg-August-University of Goettingen, Göttingen, Germany; 3 Max Planck Institute for Biophysical Chemistry, Göttingen, Germany; 4 Felix Bernstein Institute for Mathematical Statistics in the Biosciences, Göttingen, Germany

**Keywords:** Deconvolution, Flickering, Fully automatic, Hidden Markov models, Homogeneous and heterogeneous noise, Ion channel recordings, Low-pass filtering, Open-channel noise, PorB, Subconductance states

## Abstract

Analysis of patchclamp recordings is often a challenging issue. We give practical guidance how such recordings can be analyzed using the model-free multiscale idealization methodology JSMURF, JULES, and HILDE. We provide an operational manual how to use the accompanying software available as an R-package and as a graphical user interface. This includes selection of the right approach and tuning of parameters. We also discuss advantages and disadvantages of model-free approaches in comparison to hidden Markov model approaches and explain how they complement each other.

**Supplementary Information:**

The online version contains supplementary material available at 10.1007/s00249-021-01506-8.

## Introduction

The patchclamp technique has been and still is a fundamental tool for the quantitative analysis of electrophysiological processes of transmembrane proteins, in particular of ion channels (Neher and Sakmann 1976; Sakmann and Neher 1995). A detailed understanding of the dynamics of transmembrane proteins and their manifold interactions with their surrounding is of high importance in medicine and biochemistry, for instance for the development of new drugs (Kass 2005; Overington et al. 2006). However, most electrophysiologists will agree that conducting patchclamp experiments, but also the analysis of their recordings is a challenging issue, and the latter is far from being a routine data analysis in general (Sivilotti and Colquhoun 2016). In this work, we provide practical guidance on how to analyze such recordings. We focus mainly on model-free multiscale idealizations (explained below), which we have developed over the last decade.

**Fig. 1:**
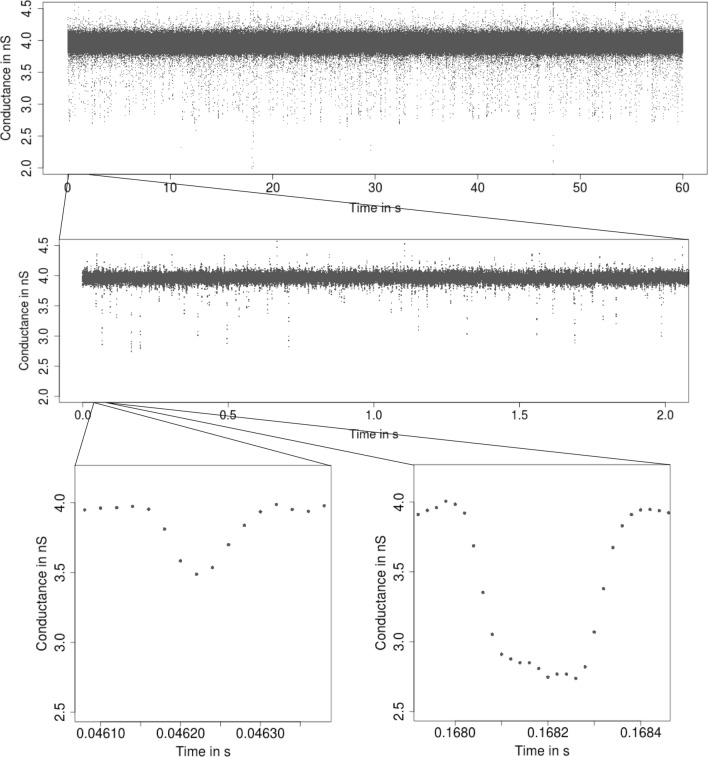
From seconds to microseconds: patchclamp recording (grey points) displayed at the level of seconds (top panel), of milliseconds (middle panel), and of microseconds (bottom panels). Data points result from a representative conductance recording of PorB wild type with 1 mM ampicillin by the patchclamp technique using black lipid membranes at 80 mV. Data points are explicitly displayed instead of a line plot, which provides an accurate representation at fine resolution levels

**Patchclamp recordings** The patchclamp technique allows one to measure the conductance of a channel (i.e., the recorded current divided by the applied voltage) over time. An example is given in Fig. 1. It shows a recording of the outer membrane porin PorB from *Neisseria meningitidis*, a pathogenic bacterium in the human nose and throat region (Virji 2009). PorB is a trimeric porin and the second most abundant protein in the outer membrane of *Neisseria meningitidis*. The added antibiotic ampicillin blocks the ion flow for short periods of time which allows one to draw conclusions about the transport of antibiotics into the cell, which is relevant for the understanding of antibiotic resistances. For further details, see (Bartsch et al. 2019, 2020). In addition, we will also use a PorB dataset without ampicillin (Fig. 6) and a Gramicidin A dataset (Fig. 4) throughout this work as illustrating examples.^1^

**Idealization** Important dynamics such as the number of conductance levels, their values, and how long each level persists can be examined provided the conductance recordings (data points) are properly idealized (Colquhoun 1987; Sakmann and Neher 1995), i.e., the conductance trace over time (the underlying signal) is accurately reconstructed (estimated, denoised).

An idealization can either be obtained model free^2^, i.e., without prior assumptions about the gating dynamics, or in a model-based way by assuming an underlying statistical (parametric) model with a few parameters for the gating dynamics. For the latter, most commonly hidden Markov models (HMMs) are used, see (Ball and Rice 1992) for an early reference, where parameters correspond to states, transition probabilities, and noise characteristics.

**Fig. 2:**
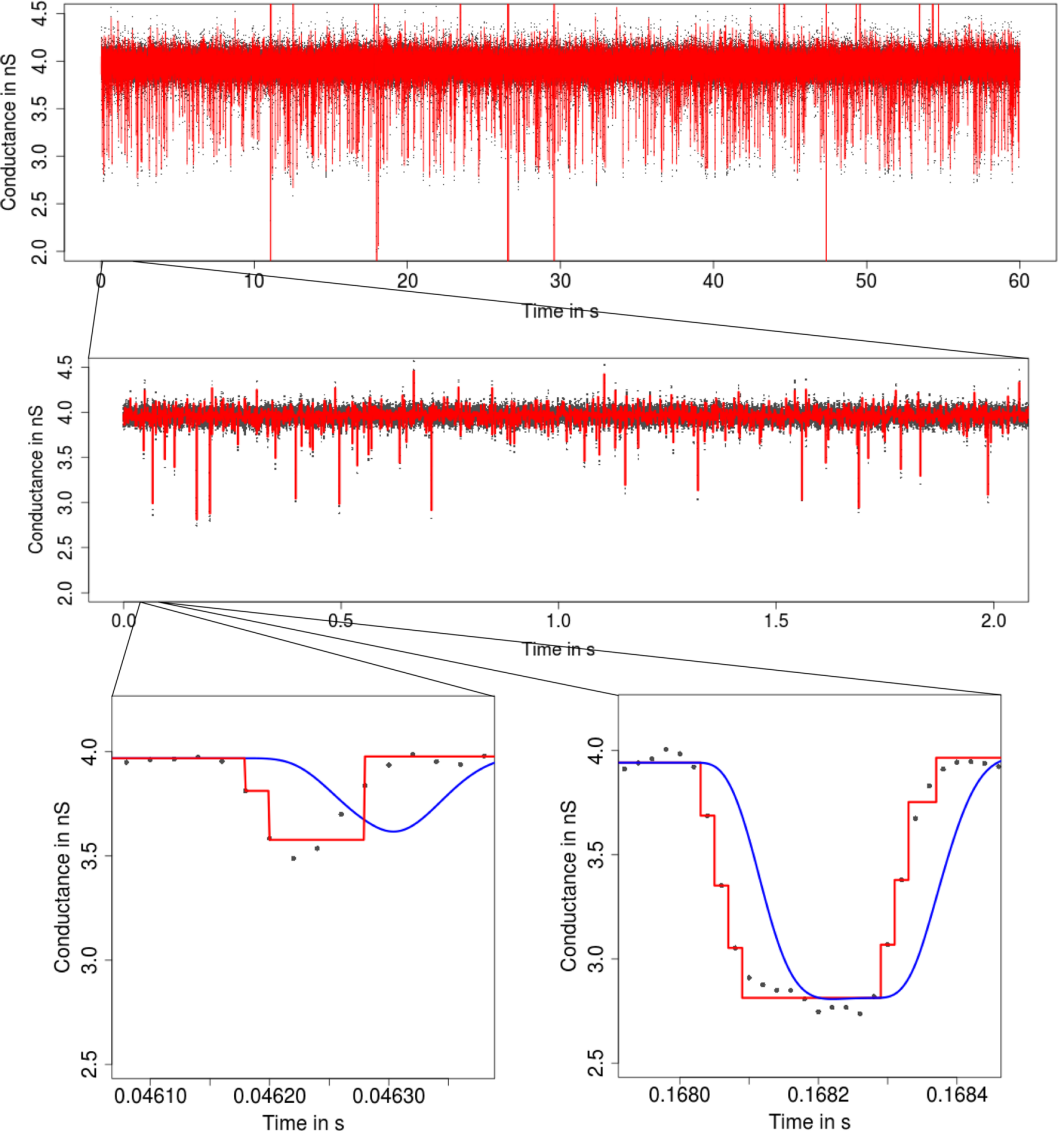
Idealization (red) of the observations in Fig. 1 by ${\text {SMUCE}}$ (Frick et al. 2014) displayed on three different temporal scales. In the lower panels, we also show the convolution of the idealization with the low-pass filter (blue). ${\text {SMUCE}}$ is a multiscale method similar to our model-free idealization approaches, but does *not* take into account filtering. Hence, local dependencies due to colored noise are misinterpreted as events. Moreover, abrupt conductance changes are split into multiple steps, since the convolution of the conductance with the low-pass filter is ignored. Consequently, ${\text {SMUCE}}$ overestimates massively the number of conductance changes

**Filtering** The noise before filtering is often assumed to be Gaussian white noise. However, low-pass filters are usually integrated in the hardware of the measurement device to stay in the transmission range of the amplifier. Such filtering introduces colored noise and smooths the underlying conductance, see Fig. 12 in “Review of existing model-free idealization approaches”. Ignoring filtering typically results in the detection of false positives (additional wrong events). This is illustrated in Fig. 2, where we used ${\text {SMUCE}}$ (Frick et al. 2014), a multiscale method that does not include filtering, but is otherwise similar in spirit to our model-free idealization approaches, to be explained later. Filtering especially affects short temporal scales (at and below the magnitude of the filter length, say) and is therefore particularly relevant to the analysis of short events, also called *flickering*.

**Fig. 3:**
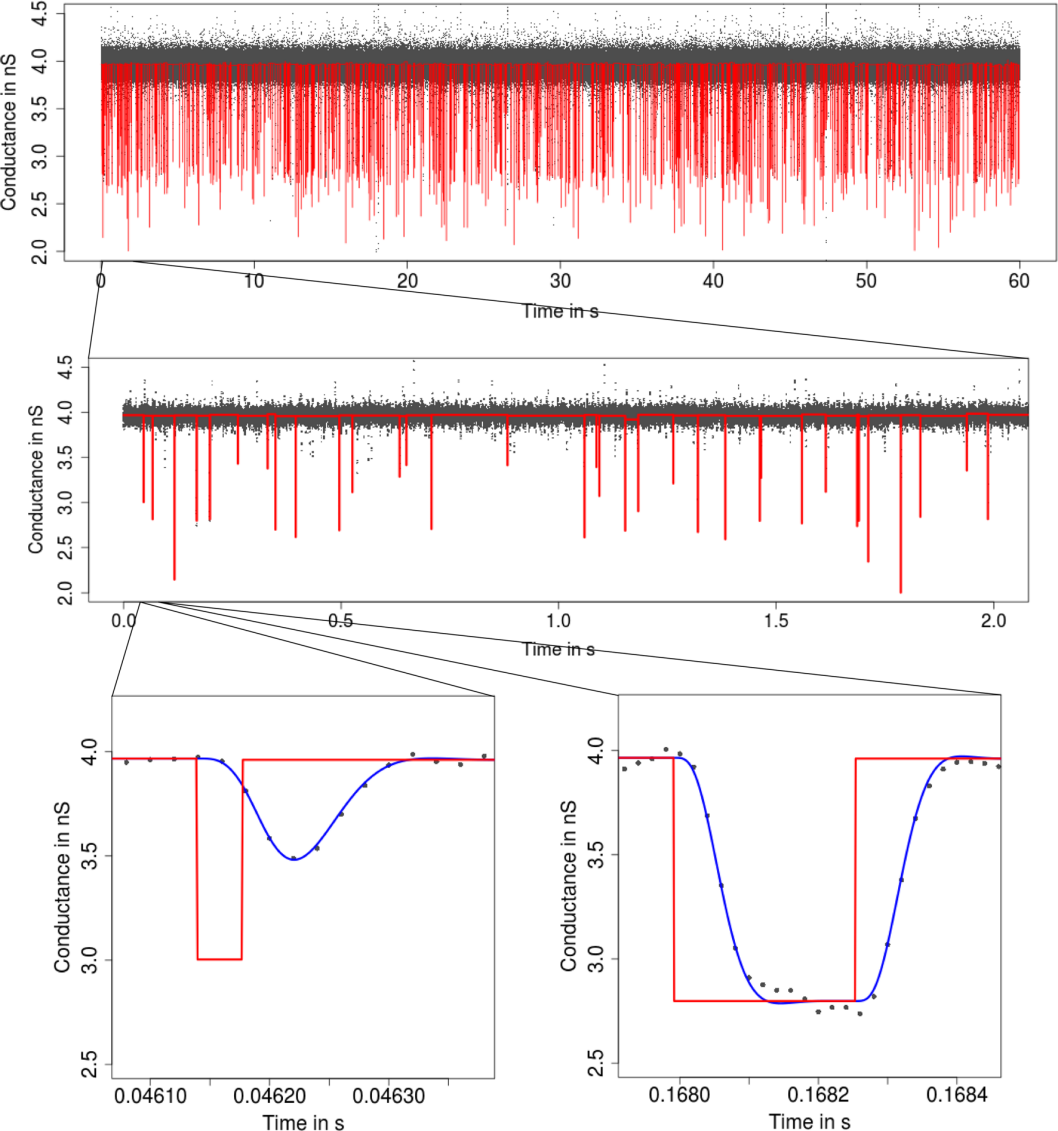
Idealization (red) of the observations in Fig. 1 by ${\text {HILDE}}$ (Pein et al. 2021) displayed on three different temporal scales. Lower panels: convolution of the idealization with the low-pass filter (blue). Events are well idealized down to microseconds

**Fig. 4:**
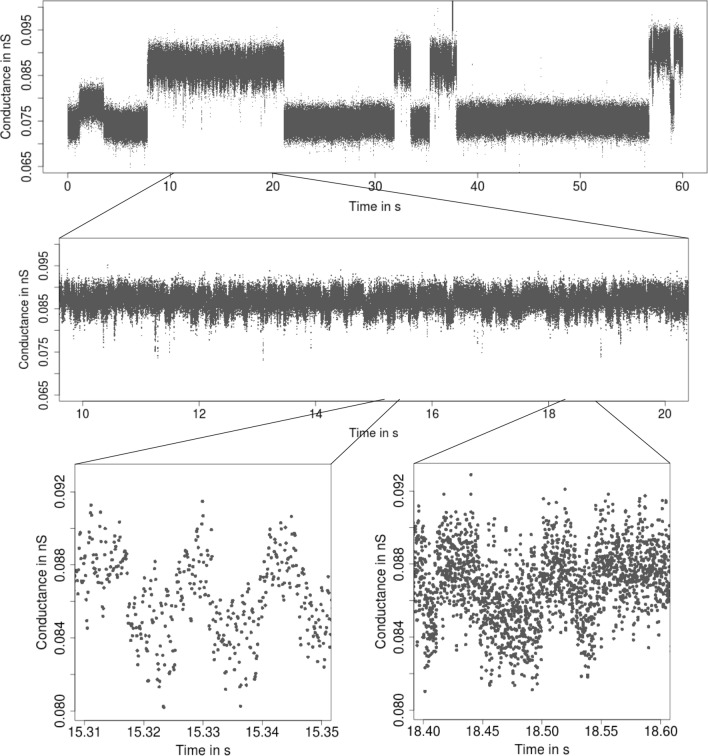
From seconds to milliseconds: patchclamp recording (grey points) displayed at the level of seconds (top panel and middle panel) and of milliseconds (bottom panels). Data points result from a representative conductance recording of an acylated gramicidin A derivative by the patch clamp technique using solvent-free bilayers at 100 mV. Small conductance changes, which most likely result from subconductance states, occur frequently

**Fig. 5:**
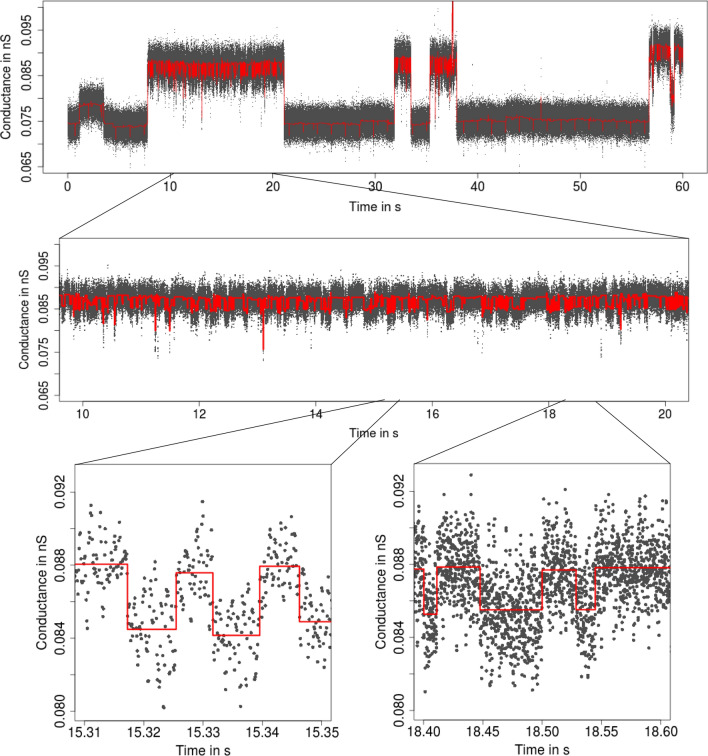
Idealization (red) of the observations in Fig. 4 by ${\text {JSMURF}}$ (Hotz et al. 2013) displayed on three different temporal scales. It idealizes the observations well and finds in particular a larger number of gating events with a small conductance change (subconductance states)

**Flickering and subgating** Flickering typically has its own dynamics and can result from various molecular processes like conformational changes of the protein (Grosse et al. 2014) or by the passage of larger molecules blocking the ions’ pathway through the protein (Raj Singh et al. 2012). An example for the latter is the PorB analysis in Figs. 1 and 3. A second potential challenge in the analysis is *subconductance* states (Fox 1987), meaning that two or more conductance levels are close to each other, as illustrated in Figs. 4 and 5.

**Model-free idealizations** In this paper, we discuss mainly our model-free idealization methods ${\text {JSMURF}}$ (Hotz et al. 2013), ${\text {JULES}}$ (Pein et al. 2018), and ${\text {HILDE}}$ (Pein et al. 2021), which are primarily designed as versatile tools to analyze patchclamp recordings in a multiscale fashion, for instance to deal well with *subconductance* states and *flickering*. Due to their multiscale character, they act on various temporal scales simultaneously and hence are able to idealize events of different lengths well in a single step. Moreover, all parameters, i.e., locations of conductance changes and conductance levels, are obtained by (local) deconvolution, and hence, they take into account low-pass filtering explicitly. Furthermore, all three approaches control the overestimation of the number of conductance changes. More precisely, the probability to detect at least one false positive is bounded approximately by the error level $\alpha$, a tuning parameter. A more-detailed review of these and further model-free idealization approaches is given in  “Review of existing model-free idealization approaches”.

All three methods can be used when homogeneous noise is assumed (i.e., the error variability does not change over time, see “Models” for details), but ${\text {JSMURF}}$ and ${\text {HILDE}}$ in addition allow for heterogeneous noise. The latter means that different parts of the data, for instance different states, may have different noise levels, as for instance caused by open-channel noise, i.e., larger noise on segments with a larger conductance. Moreover, ${\text {JSMURF}}$ requires that events are slightly longer, while ${\text {JULES}}$ or ${\text {HILDE}}$ are able to deal with flickering (short events) at the possible expense of longer computational time. Table 1 summarizes for which datasets which one of them is most suitable and  “Choosing the right method” explains those choices in full detail.

**Table 1:** Selection of the right model-free idealization method

	Homogeneous noise	Heterogeneous noise
No relevant short events exist	JSMURF (homogeneous noise)	JSMURF (heterogeneous noise)
Relevant short events exist	JULES or HILDE (homogeneous noise)	HILDE (heterogeneous noise)

For more details, see “Choosing the right method”

**Software** ${\text {JSMURF}}$, ${\text {JULES}}$, and ${\text {HILDE}}$ are implemented as R functions in the package *clampSeg* (Pein and Aspelmeier 2020). In “Using our software”, use of those methods is demonstrated. They can be combined with the packages *readABF* (Syekirin and Pein 2020) to load recordings, and *lowpassFilter* (Pein et al. 2020) for certain data processing steps around filtering such as computing the convolution of an idealization with the kernel of a low-pass filter.

Alternatively, a graphical user interface, available at https://github.com/FlorianPein/clampSegGUI together with detailed manuals on how to install it and on how to use it, allows access without requiring any R or other programming knowledge. The idealizations can be visualized in the interface, but also saved as *csv* files and hence postprocessed by any other program.

**Interplay between model-free idealizations and hidden Markov models** There is wide agreement that, except in few counterexamples (Fuliński et al. 1998; Mercik and Weron 2001; Goychuk et al. 2005; Shelley et al. 2010), the gating dynamics of ion and many other channels are usually Markovian. Hence, hidden Markov model (HMM)-based approaches are widely used to analyze patchclamp recordings. However, assuming an HMM is not only saying that the hidden states follow a Markov model, it also fixes a data generating process conditioned on the hidden states. From our own experience, we stress that this second step is usually the critical part of the assumption of an HMM. Standard (homogeneous) HMMs, where observations conditioned on the hidden Markov states are modeled as independent Gaussian observations with state-dependent expectations and variances, are often violated and commonly lead to invalid reconstructions. This is because of artifacts, which are, for instance, caused by the electronics, external vibrations, or small holes in the membrane, or because of additional high-frequency $f^2$ (violet) and long-tailed 1/*f* (pink) noise components, see for instance (Neher and Sakmann 1976; Venkataramanan et al. 1998; Levis and Rae 1993). Hence, HMM-based analyses often rely on intensive preprocessing or on more complicated models: for instance, (Venkataramanan et al. 1998) assumed an HMM that allows additional colored noise, and (Diehn et al. 2019) provided modifications to incorporate inhomogeneous errors. Moreover, low-pass filtering often requires further, computationally demanding extensions, see for instance (Venkataramanan et al. 1998; de Gunst et al. 2001; Diehn 2017; Almanjahie et al. 2019).

In contrast, model-free idealizations do not assume a specific (parametric) model for the gating dynamics and immediately provide an idealization without such an assumption. Moreover, they usually act rather locally on the data, i.e., at every location, the idealization is not influenced significantly by observations far away. Thus, they are typically more robust to artifacts and hence require often no or less pre-processing. Contrarily, HMM-based approaches have (potentially) a finer time resolution and provide more concise results. A more-detailed review of HMM-based analyses, their advantages and disadvantages in comparison to model-free approaches, and their interplay is given in “Hidden Markov Models”.

Model-free idealizations allow a flexible analysis of the number of conductance levels, and their values and which transitions are possible. To this end, one has to cluster the estimated conductance values, e.g., by fitting a Gaussian mixture distribution and assigning each value to the nearest mean value. The outcome will then have only a small number of conductance levels. This can be used to select and verify a Markov model and to estimate its parameters, which often requires taking into account missing of short events. Further details and tools which can be used for those steps are described in “Analysis of patchclamp recordings”. Finally, model-free idealizations can be used to assist HMM-based approaches in any of their analysis steps, e.g., they can be used to remove artifacts, to select and verify a specific Markov model, to provide starting values for iterative procedures such as the Baum–Welch algorithm, and to verify estimated parameters and the provided idealization.

All in all, model-free and HMM approaches have different strengths and weaknesses and hence should less be seen as competing approaches, but rather as tools that benefit from and complement each other. In fact, as an indication of a proper data analysis, it can be checked whether the results of model-free and HMM-based analyses are in compliance.

**Organization of this work** In Model-free idealizations, we give detailed instructions how to use our methods to obtain model-free idealizations. In “Models”, we provide details of the statistical models underlying the presented model-free idealization methodology. In Review of tools to analyze patchclamp recordings, we review in more detail existing approaches for the analysis of patchclamp recordings. We start in “Hidden Markov Models” with a review of HMM-based methodology, their advantages and disadvantages in comparison to model-free approaches, and the interplay of model-free idealization with them. Afterwards, in “Review of existing model-free idealization approaches”, we review some existing model-free idealization methods, with a particular focus on our approaches ${\text {JSMURF}}$, ${\text {JULES}}$, and ${\text {HILDE}}$. This is complemented by a brief summary of simulation results. Finally, in “Analysis of patchclamp recordings”, we discuss how (model-free) idealizations can be used to analyze patchclamp recordings. The paper concludes with a discussion in Discussion, in which we highlight open research questions.

## Model-free idealizations

This section provides a comprehensive guide on how to use our methods ${\text {JSMURF}}$, ${\text {JULES}}$, and ${\text {HILDE}}$, which have different strengths and weaknesses depending on certain structural features of the measured data. We explain in detail how to use our software (Using our software) and provide guidance for which method is preferable in which situations (“Choosing the right method”).

### Using our software

For this section, we use R code^3^. A tutorial similar to the one in this section is available in the supplement as a *zip* file. It contains R code, resulting figures, and the obtained fits. Hence, users are able to test whether they obtain the same results. We start by describing how recordings can be loaded, how the low-pass filter can be specified, and how our methods can be called. To this end, we require the R packages *readABF* (Syekirin and Pein 2020), *lowpassFilter* (Pein et al. 2020), and *clampSeg* (Pein and Aspelmeier 2020). All three packages are available on CRAN^4^. For users who are not familiar with R, we also provide a graphical user interface^5^, which contains detailed manuals on how to install it and on how to use it. The current guide is also available in the supplement.

**Loading the data** Patchclamp recordings are typically stored as *abf* files. The *readABF* package allows one to read such files in R. After the data set is loaded by calling *readABF*, we use *as.data.frame* to transform the data into a *data.frame* with two columns: time and conductance (the current divided by the voltage channel). We stress that this call is data set specific, since common measuring devices have a wide range of different formats and offer some freedom which channels are recorded. Additionally, users might want to work with the current instead of the conductance. Those options are described in the help file of *as.data.frame*.
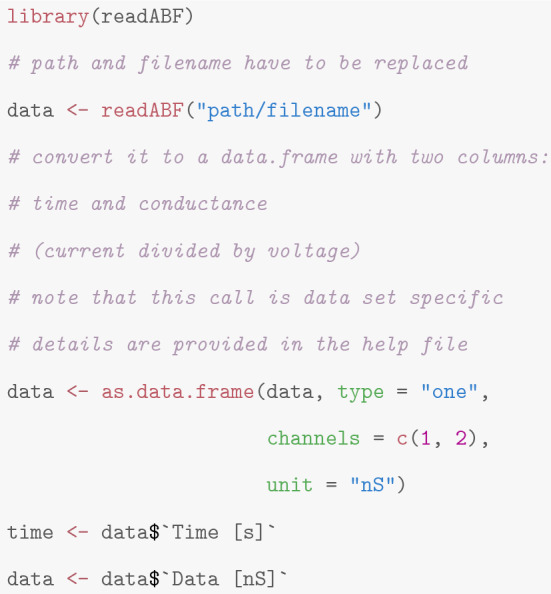


Our idealization methods but also any other approach should not be used as a black box. We strongly recommend to start with an empirical and visual data analysis to gain understanding of the datasets and major features that can be used to direct further analysis. In alignment with the underlying multiscale philosophy of our methods, we recommend always to plot on various temporal scales. See Fig. 1 for an example of such plots on three different scales ranging from a minute to milliseconds. Moreover, histograms of the raw data (point amplitude histograms), see for instance Fig. 13a and for code the paragraph ’Interpreting, plotting and verification of the output’ below, are helpful visual cues. As detailed below, this can already help to decide whether the noise is homogeneous or heterogeneous and whether short events occur in the dataset. Moreover, we recommend to identify potential artifacts that might disturb analysis and interpretation. However, we have found that model-free idealizations are usually quite robust to artifacts. Hence, our default suggestion is first to apply the idealization methods on the unmodified dataset and to decide later whether artifacts require a more careful analysis.

**Low-pass filter **Our methodology requires to specify correctly the low-pass filter in the measurement device. The type, often a Bessel filter with an even number of poles, should be specified in the hardware documentation. The sampling rate and cut-off frequency can typically be varied by the user. In our example (Fig. 1), the recordings were sampled at 50,000 Hz and low-pass filtered by a 4-pole Bessel filter with normalized cut-off frequency of 0.1 (5,000 Hz cut-off frequency in time domain).

For simplification and since the error is negligible, we truncate the kernel of the low-pass filter after *m* data points, for sufficiently large *m*. As a working rule, we choose *m*, such that the autocorrelation function of the untruncated analogue low-pass filter is below $10^{-3}$ afterwards, which leads for instance to $m = 11$ in the example above.

This is implemented in the function *lowpassFilter* in the package *lowpassFilter* (Pein et al. 2020) (currently only Bessel filters are supported). The following code creates the filter object.
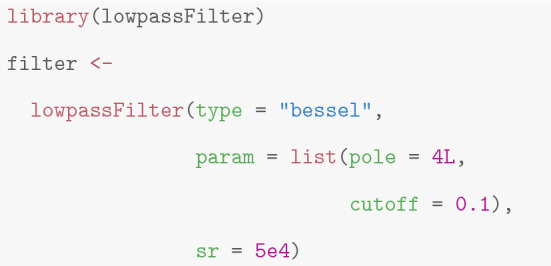


We strongly recommend to verify that the filter is correctly specified by zooming into single events and checking whether the obtained idealization convolved with the low-pass filter fits the observations well. This is detailed in paragraph ’Interpreting, verification and storing of the output’ below, where we discuss in more generality how to assess the quality of an obtained idealization. Additionally, one can compare the auto-correlation resulting from the filter, *filter$acf*, with the estimated auto-correlation of the recordings. To this end, one can either apply standard time-series estimators, as, for instance, offered by the *acf* function in R, to long segments without conductance changes or use the robust difference-based estimators of (Tecuapetla-Gómez and Munk 2017) on the raw data, available in the R-package *dbacf*^6^.

**Obtaining an idealization** ${\text {JSMURF}}$, ${\text {JULES}}$, and ${\text {HILDE}}$ are available in the package *clampSeg*. All three functions can be applied when homogeneous noise is assumed, but only ${\text {JSMURF}}$ and ${\text {HILDE}}$ allow for heterogeneous noise. The following code illustrates how to call those functions depending on whether the noise is homogeneous or heterogeneous. See “Choosing the right method” for guidance which method and which noise option should be chosen to idealize a given measurement.
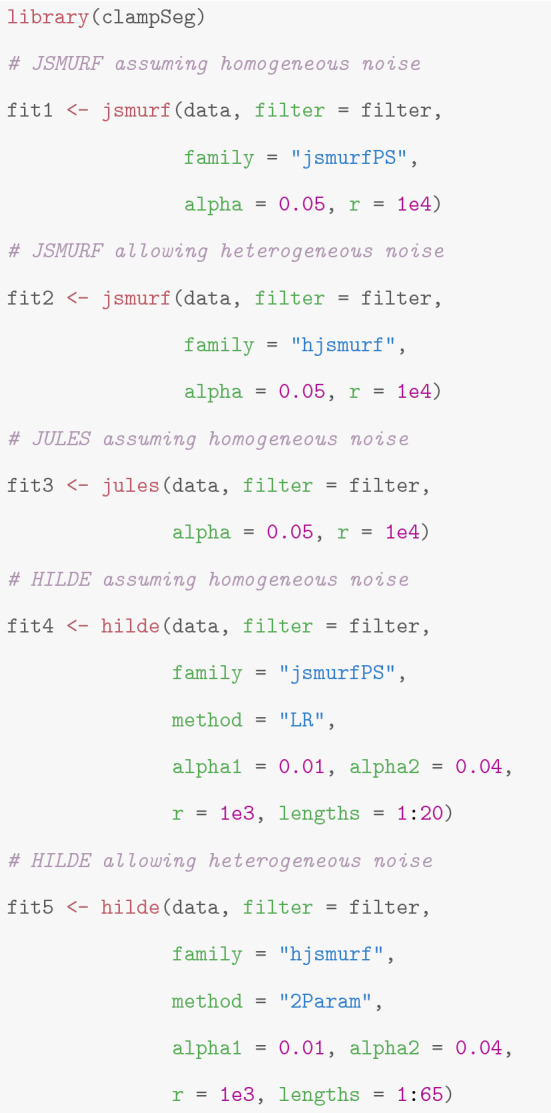


The followings paragraphs discuss run time, required Monte Carlo simulations, the output of the approaches, and how to proceed with it. Furthermore, we explain a potentially occurring warning and how to choose tuning parameters, e.g., *r* and *alpha*.

**Run time and Monte Carlo simulations** The run time of all approaches depends on the size of the dataset, but also on the number of detected events. The primary reason are Monte Carlo simulations which are required to obtain critical values that balance the probabilities of detection of true events and of false positives. Monte–Carlo simulations depend on the number of data points and on the low-pass filter. Hence, a new Monte Carlo simulation is required when new values for those parameters occur or when more repetitions are requested. Depending on the number of data points and the total number of repetitions *r*, Monte Carlo simulations may take long, even up to several hours. Hence, we store and load their results, such that they have to be performed only once and the run time will be much smaller when an idealization with the same parameter is computed. They are fully automatically stored in the workspace and on the disk of the local computing machine; for more details, see the documentation of the function *getCritVal* in the package *clampSeg* (Pein and Aspelmeier 2020). To keep track of the progress of a Monte Carlo simulation, one can set the argument *messages* to a positive integer value *m* to print a message every *m* repetitions.

While a larger number of repetitions increases the run time of the simulations, it also reduces statistical errors in the computation of the critical values. For a final analysis, we recommend to use the default values, 10,000 for ${\text {JSMURF}}$ and ${\text {JULES}}$ and 1,000 for ${\text {HILDE}}$. For a quick analysis, for instance to decide whether further measurements or analyses are required, few hundreds up to 1,000 repetitions usually suffice.

Additionally, also the main computation of the idealization can take some time, usually between few seconds and few minutes, depending on the used idealization method, on the size of the dataset and on the number of detected events. Usually, the run time increases with the complexity of the idealization approach, ${\text {JSMURF}}$ is the fastest, and ${\text {HILDE}}$ the slowest. A situation which is computationally particularly demanding is displayed in Fig. 9 (see below). ${\text {JSMURF}}$ detects almost no events. Due to internals in the dynamic programming algorithm, this causes a considerably long-run time, in this example of roughly half an hour. We stress that ${\text {HILDE}}$ uses ${\text {JSMURF}}$ as an initial step and hence also ${\text {HILDE}}$ is slow in such a situation, though it detects many events as it is able to resolve events on smaller temporal scales at and below the magnitude of the filter length.

**Interpreting, plotting, and verification of the output** All shown idealization methods return an object of the classes *stepblock* and *localDeconvolution*. We omit the exact structure of it (and refer to the man files of the called functions), but demonstrate important ways how to proceed with such an object. First of all, the idealization can be plotted using standard functions in R. Furthermore, the convolution of the idealization with the kernel of the low-pass filter can be computed using the function *getConvolution* in the package *lowpassFilter*. The following code demonstrates how to do so. It provides the lower left panel in Fig. 3.
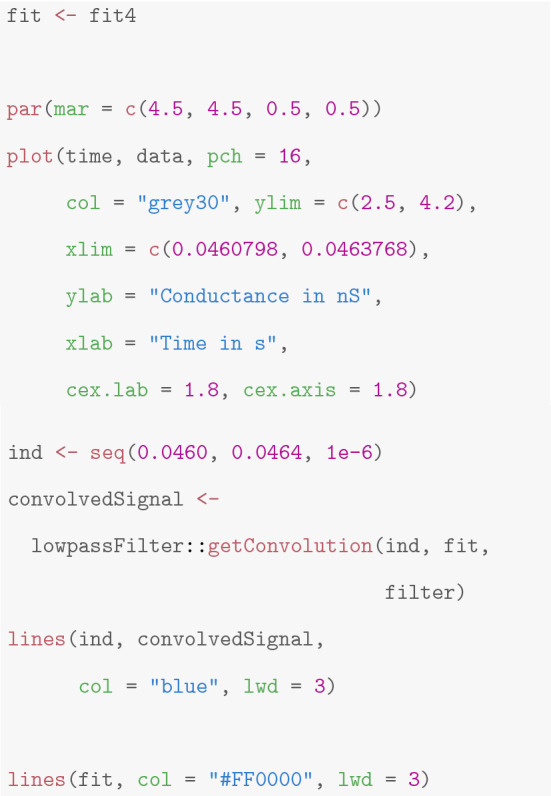


In Fig. 3, we found that the convolution fits the recorded observations well, which is a confirmation for our idealization, but also for a correct specification of the model, in particular of the underlying low-pass filter. We always recommend such a graphical inspection to evaluate the quality of the idealization. If the idealization is not sufficiently good, one might modify tuning parameters (see the paragraph below), try a different idealization method (see “Choosing the right method”), remove artifacts, or seek to improve the quality of the recordings.

Obtaining a model-free idealization is usually only one step in a data analysis. In “Analysis of patchclamp recordings”, we discuss typical follow-up steps. The idealized conductance values and the start and end times of the segments are given in *fit$values*, *fit$leftEnd*, and *fit$rightEnd*, respectively. For instance, the following code creates histograms of the raw data, often called point amplitude histogram, of the idealized conductance levels, often called event histogram, and of the amplitudes, i.e., of the differences between the idealized conductance levels. Examples are given in Fig. 13. We use the half sample mode (Robertson and Cryer 1974), implemented in the *R*-package *modeest*, to determine the underlying conductance levels, see the paragraph ’Analysis of the conductance levels’ in “Analysis of patchclamp recordings” for further discussion.
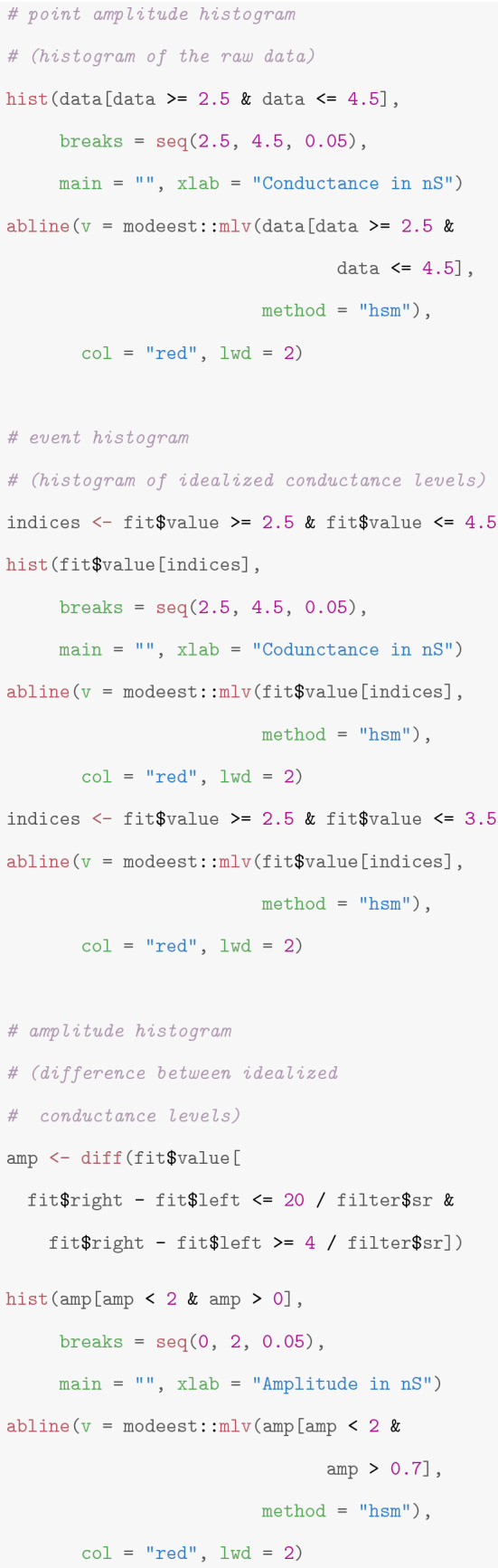


**Warning** Users may experience a *warning* saying *“at least one segment could not be deconvolved since two successive short segments occurred”*. This is caused by the fact that the deconvolution approach incorporated in our methods can only deal with single changes or with isolated peaks (two changes in quick succession but separated by few more observations from other events). Obtaining a deconvolution for three or more changes in quick succession is complicated and time-consuming, and hence, we decided to ignore such events when applying a deconvolution, but to mark them in *attr(fit, "noDeconvolution")*. For a further analysis, we usually recommend to ignore such events as they might even indicate artifacts. This can be done by setting all marked values to *NA*.
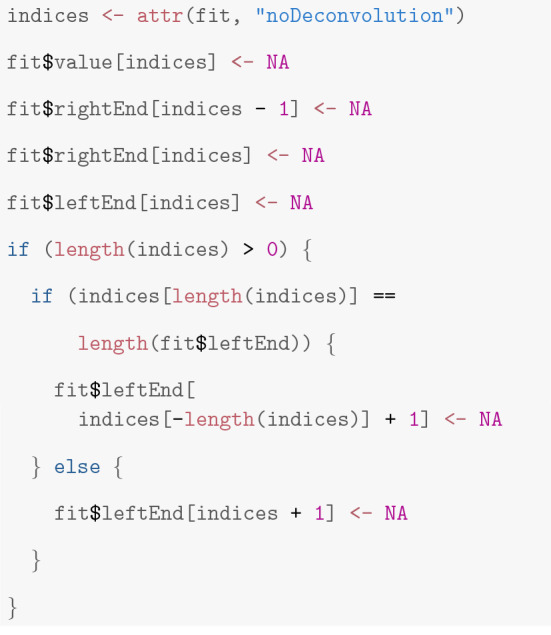


If too many segments are marked and they appear to be important for the given dataset, we cannot recommend to use our approaches, in this situation of *extreme/high flickering* a better alternative might be approaches based on conductance distribution fitting; for further details, see our review in “Hidden Markov Models”.

**Storing of the output** To allow proceeding in a different program, one can store the idealization for instance in a *csv* file as demonstrated by the following code. Note that we also remove the first and last segment, since their true start and end, respectively, cannot be identified by the data.
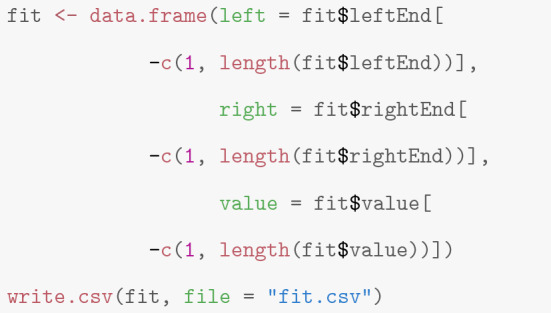


**Tuning parameters** All three methods have multiple parameters which can be tuned to adapt to particular needs. Nonetheless, it is advisable to leave them unchanged unless specific reasons exists. All parameters are described in the man files of the called functions and in the referenced papers. Hence, in the following, we will only give a brief overview about the most important ones. Further details are also provided in the review of our idealization approaches in “Review of existing model-free idealization approaches”.

The choice of the number of repetitions of the Monte Carlo simulations, the argument *r*, was already discussed above in paragraph ’Run time and Monte Carlo simulations’. The parameters *alpha*, *alpha1*, and *alpha2* are error levels $\alpha , \alpha _1, \alpha _2$ that bound approximately the probability of detecting one or more false positives (under the idealized scenario that the observations follow exactly the assumed model). As a default choice, we suggest $\alpha = 0.05$. Larger values increase the chance to detect true events, but also to detect more false positives. One may use larger $\alpha$ values to ’screen’ if important events are difficult to detect.

For ${\text {HILDE}}$, the error level $\alpha := \alpha _1 + \alpha _2$ is split between the multiscale criterion of ${\text {JSMURF}}$ (error level $\alpha _1$) and the local tests (error level $\alpha _2$). As default values, we suggest $\alpha _1=0.01$ and $\alpha _2=0.04$, since the focus of ${\text {HILDE}}$ is typically on detecting short events primarily, while events on larger scales are often easier to detect. More weight can be put on $\alpha _1$ if either short events are of less interest or if long events are difficult to detect, as well, e.g., since they have a smaller jump size than the short events, for instance because of subconductance states. ${\text {HILDE}}$ requires to specify the largest scale $l_{\max }$, this value should be chosen, such that all events on larger scales are reliably detected by ${\text {JSMURF}}$. If required, this can be tested by applying ${\text {JSMURF}}$ or by Monte Carlo simulations. In our R code, see the example code in the paragraph ’Obtaining an idealization’ above, one can specify the largest scale by setting lengths $= 1:l_{\max }$. Note that the R code offers the additional flexibility to omit some scales below $l_{\max }$. This can be used to save run time or to increase slightly the detection power on the remaining scales.

### Choosing the right method

A guide which method to use is given in Table 1. The two main criteria are whether the noise is homogeneous or heterogeneous and whether short events are present and relevant. Recall that ${\text {JSMURF}}$, ${\text {JULES}}$, and ${\text {HILDE}}$ are all suitable when one assumes homogeneous noise, but only ${\text {JULES}}$ and ${\text {HILDE}}$ allow for heterogeneous noise. Moreover, ${\text {JULES}}$ and ${\text {HILDE}}$ are designed to deal with short events, while ${\text {JSMURF}}$ requires that events are slightly longer. Because of run time and precision, we generally recommend to use the simplest approach that is suitable for a dataset. Unless the dataset demands otherwise, we recommend ${\text {JSMURF}}$ over ${\text {JULES}}$ over ${\text {HILDE}}$ and a homogeneous over a heterogeneous noise setting.

**Fig. 6:**
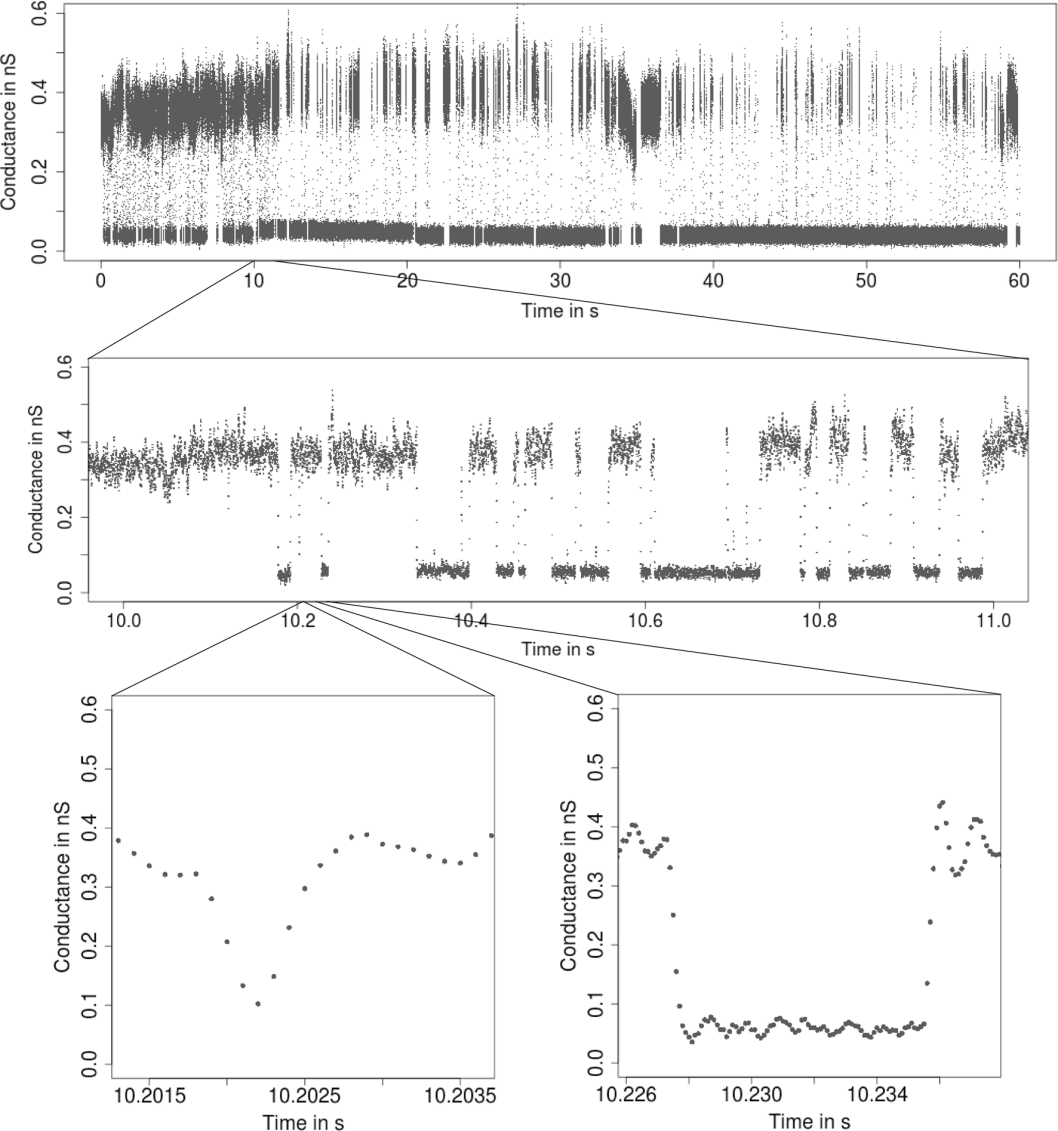
From seconds to microseconds: patchclamp recording (grey points) displayed at the level of seconds (top panel), of milliseconds (middle panel), and of microseconds (bottom panels). Data points result from a representative conductance recording of PorB by the patch clamp technique using solvent-free bilayers at 20 mV. The observations in the open state have visibly a larger noise level than the ones in closed state

**Visual inspection** *Homogeneous noise* means that the noise distribution is the same at all times and for all conductance levels; otherwise, the noise is called *heterogeneous*. Heterogeneous noise is often clearly visible by naked eye, as in Fig. 6 where the noise level is higher for the higher conductance level, whence one should use either ${\text {JSMURF}}$ or ${\text {HILDE}}$ with heterogeneous noise setting. In most cases, if heterogeneous noise is not clearly visible, approaches that assume homogeneous noise are suitable.

A *short event* is defined by two conductance changes in quick succession, e.g., a channel opening only very briefly before closing again. ${\text {JSMURF}}$ should be used if it is not expected to miss relevant short events. Which events are too short depends not only on the absolute length, but also on the magnitude of the conductance change, noise levels, filtering, and tuning parameters. At least, events shorter than filter length will certainly be missed by ${\text {JSMURF}}$. Figure 1 shows such an example, whence one should use either ${\text {JULES}}$ or ${\text {HILDE}}$.

**Empirical comparison** If visual inspection is not sufficient, we suggest the following empirical procedure. The user should apply all potentially suitable methods to a small excerpt of the data and decide which leads to the best idealization. In general, if the idealizations are similar, the simpler approach should be preferred.

To illustrate the procedure for short events, consider Fig. 3 where we see that ${\text {HILDE}}$ detects a large number of short events. In comparison, we see in Fig. 9 that ${\text {JSMURF}}$ is not able to detect those events and hence is unsuitable for this dataset. In this case, ${\text {HILDE}}$ appears to be more suitable. Contrarily, Fig. 5 demonstrates that ${\text {JSMURF}}$ is very suitable to idealize the Gramicidin dataset, where no short events occur, but events with small conductance changes, while ${\text {JULES}}$ struggles to detect all of them, as seen in Fig. 10, since it also searches for short events and hence has slightly less power on larger temporal scales.

**Fig. 7:**
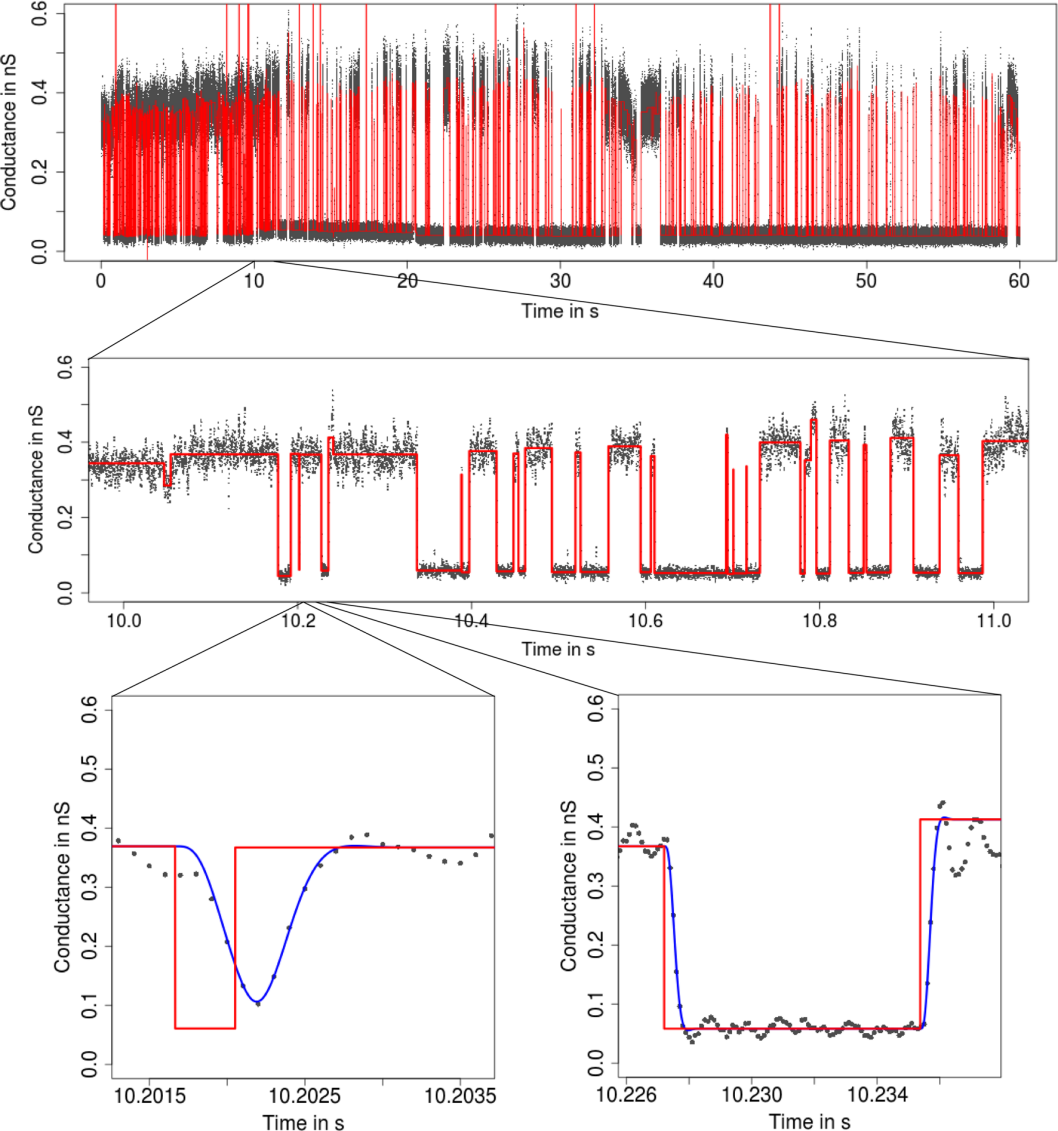
Idealization (red) of the observations in Fig. 6 by ${\text {HILDE}}$ (Pein et al. 2021) displayed on three different temporal scales. Lower panels: convolution of the idealization with the low-pass filter (blue). Events are well idealized down to very short temporal resolutions

**Fig. 8:**
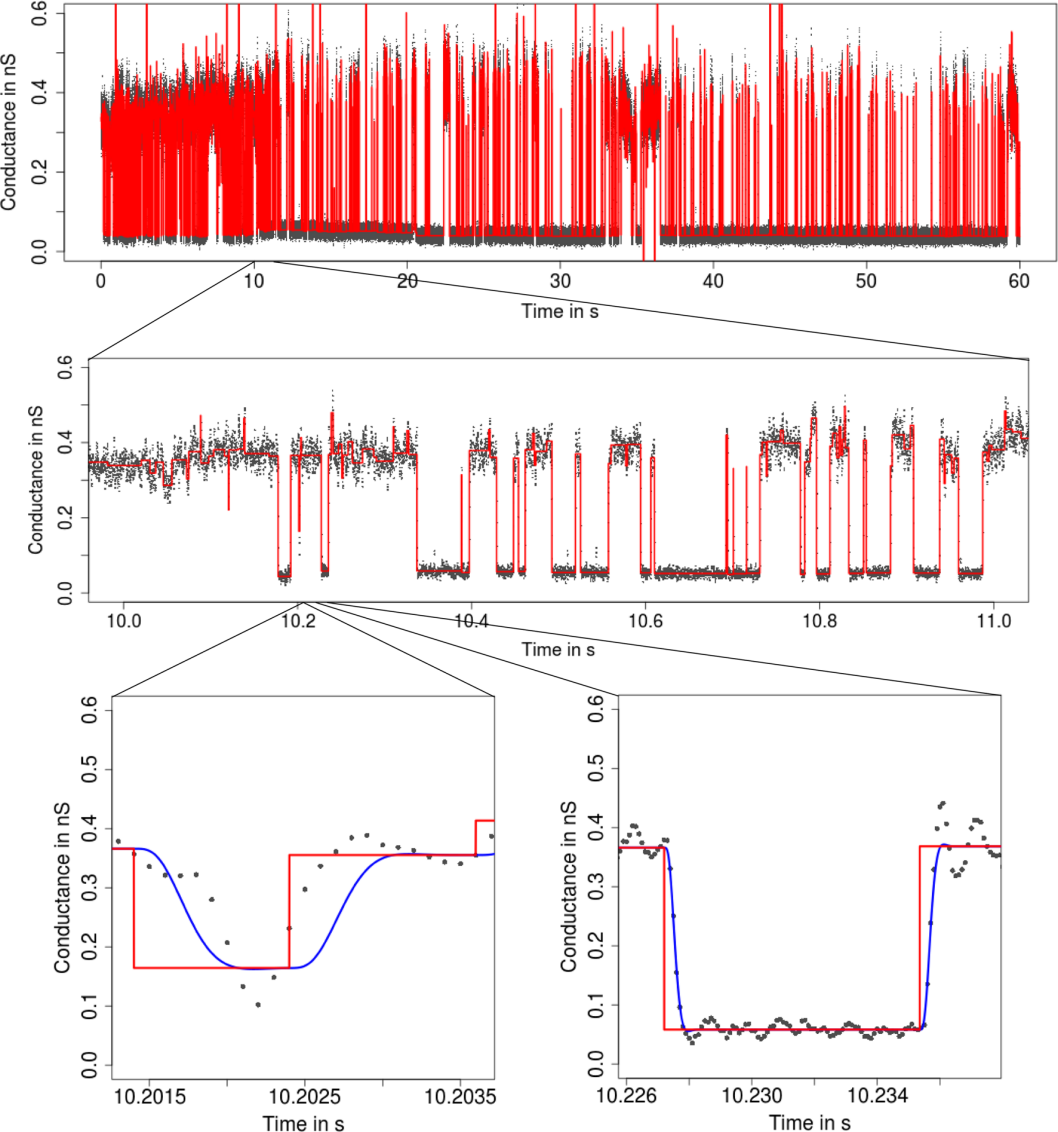
Idealization (red) of the observations in Fig. 6 by ${\text {JULES}}$ (Pein et al. 2018) displayed on three different temporal scales. Lower panels: convolution of the idealization with the low-pass filter (blue). ${\text {JULES}}$ detects short events, but finds many small events, which are most likely false positives, at areas of high conductance and high variance (see, for instance, the idealization of the observations around 0.36 nS in the middle panel). These detections prevent ${\text {JULES}}$ from performing a deconvolution at those positions (see, for instance, the lower left panel) and make the idealization unreliable

To illustrate the procedure for heterogeneous noise, we idealized the observations in Fig. 6, which have visibly heterogeneous noise, with ${\text {HILDE}}$, which is designed to deal with heterogeneous noise. Results are displayed in Fig. 7. For comparison, an idealization by ${\text {JULES}}$, assuming homogeneous noise, is displayed in Fig. 8. We see that ${\text {JULES}}$ detects many additional events in the open state, which has higher noise level, and while it is able to detect the short events, the fit is visibly worse than the fit by ${\text {HILDE}}$.

To decide whether the noise is heterogeneous, we recommend to more advanced users also the following systematic approach: if longer segments without gating events are present, one can use them to estimate the noise level. Alternatively, one can idealize the data with ${\text {JSMURF}}$ or ${\text {HILDE}}$ with heterogeneous noise setting and use the idealization to determine noise levels as detailed in (Pein et al. 2021, Section VI-C).

Finally, if homogeneous noise is assumed and short events are relevant, we usually recommend to use ${\text {JULES}}$ instead of ${\text {HILDE}}$ as it is simpler and faster. Only if events are very short, such as in Fig. 3, ${\text {HILDE}}$ should be used as it detects such events more likely.

**Fig. 9:**
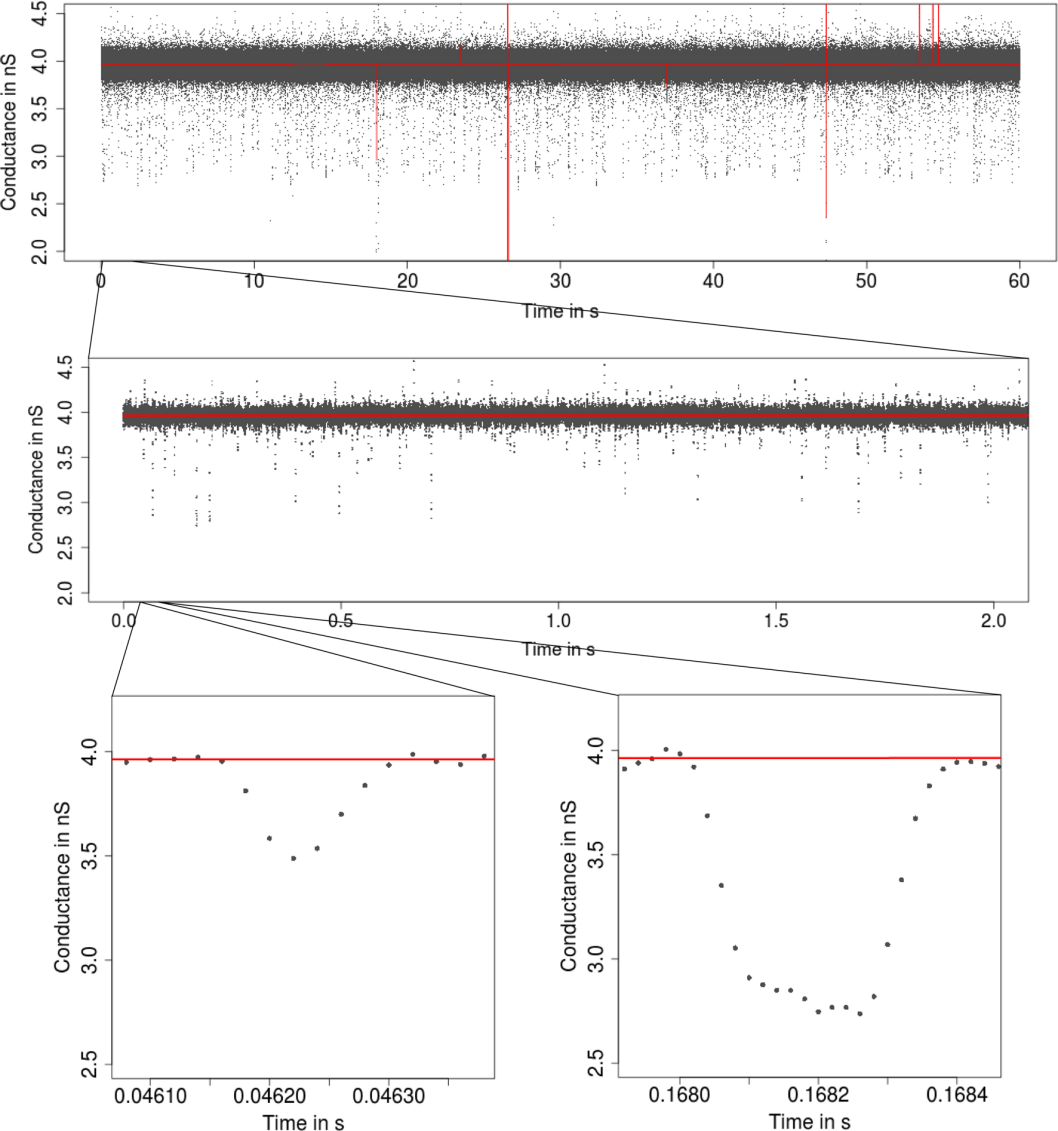
Idealization (red) of the observations in Fig. 6 by ${\text {JSMURF}}$ (Hotz et al. 2013) displayed on three different temporal scales. Almost no events are detected, since all events are around or below the magnitude of the filter length

**Fig. 10:**
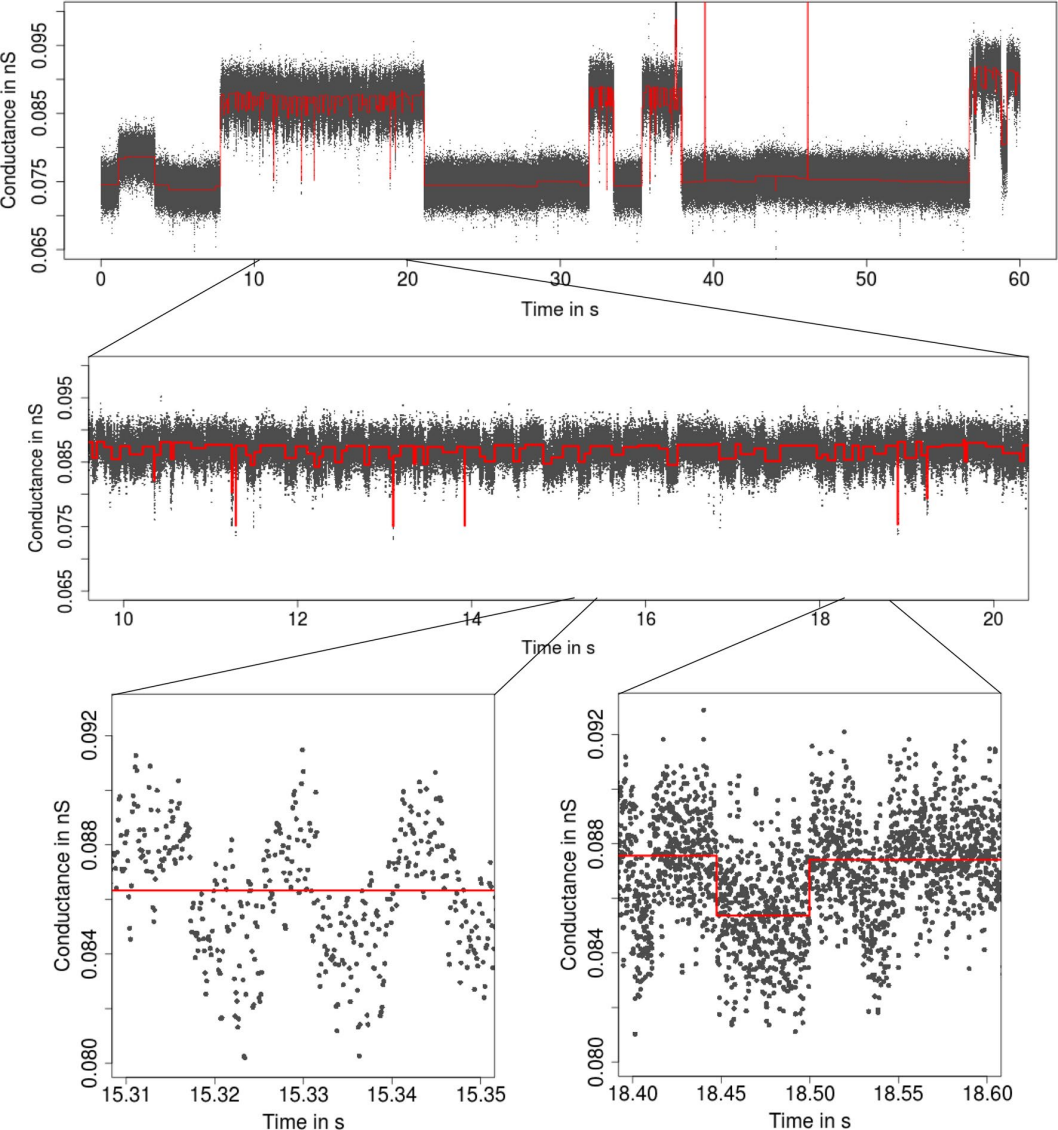
Idealization (red) of the observations in Fig. 4 by ${\text {JULES}}$ (Pein et al. 2018) displayed on three different temporal scales. It misses many of the small conductance changes, since the fact that it also looks for very short events (which are not present in this dataset) slightly decreases detection power for long events compared to ${\text {JSMURF}}$

## Models

In this section, we explain the statistical models underlying our methodology. For more details, see (Hotz et al. 2013; Pein et al. 2018, 2021).

We assume that the recorded data $Y_1,\ldots ,Y_n$ (the measured conductance at time points $t_i=i/f_{\varvec{s}},\ i = 1,\ldots ,n$, equidistantly sampled at rate $f_{\varvec{s}}$) result from a conductance *f* perturbed by a centered Gaussian white noise process $\eta$. The noise is scaled by the noise level $\sigma$. Furthermore, conductance and noise are convolved with an analogue low-pass filter, with (truncated) kernel $F_m$. Hence, after digitization at sampling rate $f_{\varvec{s}}= n / \tau _{{\text {end}}}$, we obtain:

1 $$\begin{aligned} \begin{aligned} Y_i = \big (F_m *(f + \sigma \eta )\big )\left( i/f_{\varvec{s}}\right) = (F_m *f)(i/f_{\varvec{s}}) + \epsilon _i,\\&i=1,\ldots ,n, \end{aligned} \end{aligned}$$

with $*$ the convolution operator. Here, *n* denotes the total number of data points (typically several hundred thousands up to few millions). Hence, the resulting errors $\epsilon _1,\ldots ,\epsilon _n$ are Gaussian and centered, but correlated (colored noise).

The conductance *f* is assumed to be piecewise constant with potentially many different (unknown) segments of (unknown) length and size. The noise can either be homogeneous, i.e., the noise level $\sigma$ does not vary over time, or heterogeneous. In the latter case, we assume the noise level $\sigma$ to be an unknown piecewise constant function with potential jumps at the locations where the conductance changes, since changes of the noise level also depend on gating events^7^. More precisely, we model the conductance *f* and the noise level $\sigma$ by:

2 $$\begin{aligned} \begin{aligned} f(t)&= \sum _{j=0}^{K}\,c_j\,{\mathbb{1}}_{[\tau _j, \tau _{j+1})}(t) \text { and } \sigma \equiv \sigma _0\in \mathbb {R},\\&\quad \text {if homogeneous noise is assumed},\\ f(t)&= \sum _{j=0}^{K}\,c_j\,{\mathbb{1}}_{[\tau _j, \tau _{j+1})}(t) \text { and } \sigma (t) = \sum _{k=0}^{K}\,s_k\,{\mathbb{1}}_{[\tau _k, \tau _{k+1})}(t),\\&\quad \text {if heterogeneous noise is assumed}, \end{aligned} \end{aligned}$$

where *t* denotes physical time. The (unknown) conductance levels are denoted as $c_0, \ldots , c_K$, the (unknown) noise levels as $s_0,\ldots ,s_K>0$, the (unknown) number of gating event as *K*, and the (unknown) locations of the gating events as $-\infty =: \tau _0< \tau _1< \cdots< \tau _K < \tau _{K+1}:=\tau _{{\text {end}}}$. We stress that the class of signals in (2) is very flexible as potentially any arbitrary number of gating events at arbitrary conductance levels and arbitrary noise levels can be imposed, see Fig. 3 for an example.

## Review of tools to analyze patchclamp recordings

**Fig. 11:**
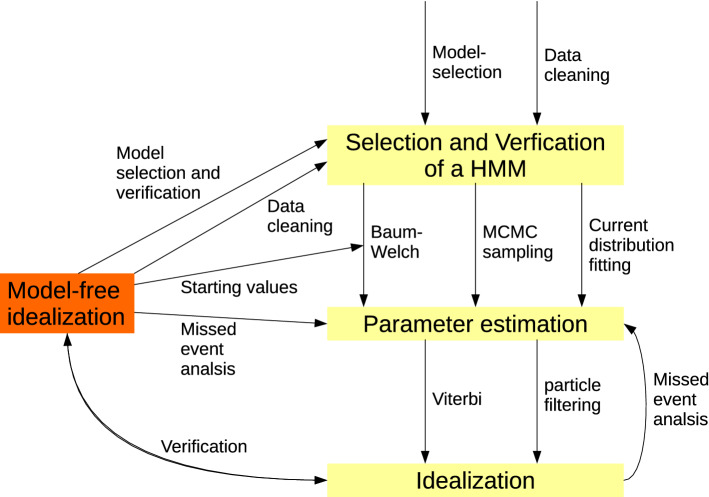
Illustration of the interplay between HMM and model-free approaches

In this section, we give a review about methods for the analysis of patchclamp recordings. We start in Hidden Markov Models with a HMM-based analysis and discuss also their interplay with model-free idealizations as well as their advantages and disadvantages in comparison to model-free approaches. The analysis by and the interplay between the different approaches is also illustrated in Fig. 11. Second, we review existing model-free idealization methods in Review of existing model-free idealization approaches. Finally, we discuss in Analysis of patchclamp recordings how idealizations can be used to analyze patchclamp recordings. Given the large amount of different methodology, we are by far not able to give a full review. The following only intends to summarize major ideas to help the reader to put ${\text {JSMURF}}$, ${\text {JULES}}$, and ${\text {HILDE}}$ in the right context.

### Hidden Markov models

**HMM-based analysis** We limit our discussion mostly to homogeneous HMMs, which means that the parameters, which describe state transition properties and noise distribution, are constant in time. Inhomogeneous HMMs, see, for instance, (Diehn et al. 2019), are rarely used, as they are computationally more challenging and theoretical guarantees for parameter estimates are much harder to prove. As already discussed in the introduction, the assumption of a homogeneous Markov chain underlying the gating dynamics is almost always appropriate, but the assumption of a homogeneous error distribution to obtain a homogeneous HMM is more critical, since, e.g., because of artifacts, often intensive data cleaning or more complicated models are required. We stress that the quality of an HMM-based analysis crucially depends on the stringent modeling assumption given by a HMM.

Obtaining an idealization by an HMM proceeds in several steps (illustrated in the right-hand side of Fig. 11): First, a specific hidden Markov model has to be selected and ideally verified. This includes to find a Markov model for the gating dynamics, e.g., to fix the number of states and which transitions are possible. Note, that often multiple Markov states are required for one conductance level, e.g., to accommodate different noise levels or dwell times. Though data-driven model-selection tools are available, see, e.g., (Gassiat and Keribin 2000; Gassiat and Boucheron 2003; Celeux and Durand 2008; Chambaz et al. 2009; Lehéricy 2019) and the references therein, this is often done manually by an empirical data analysis or by repeating the steps below until results are satisfying, which can be time-consuming and introduces subjectivity.

As soon as a specific HMM is selected, parameters of the Markov model can either be estimated by the Baum–Welch algorithm, see (Venkataramanan et al. 2000; Qin et al. 2000), by Bayesian approaches, in particular MCMC sampling, see (de Gunst et al. 2001; Siekmann et al. 2011), or by approaches based on the conductance (current) distribution, see (Yellen 1984; Heinemann and Sigworth 1991; Schroeder 2015) and the references therein.

Finally, an idealization can be obtained by the Viterbi algorithm (Viterbi 1967) or by Bayesian methods, in particular particle filtering, see (Fearnhead and Künsch 2018) and the references therein. Recently, a deep neural network approach has been proposed (Celik et al. 2020), which skips the parameter estimation step and directly obtains an idealization. This approach can be seen as a hybrid method in between parametric and model-free approaches. It does not require a specific HMM to obtain an idealization, but training in advance is required, which was done by assuming classes of hidden Markov models with hyperparameters.

Once an idealization is obtained, it can be used in reverse to estimate the parameters of the Markov model. We postpone details to Analysis of patchclamp recordings, since one proceeds as for model-free idealization. Using a layered HMM on simulated filtered signals (Pein et al. 2021, Sect. IV–D) as well as in real data applications (Pein et al. 2021, Sect. V) (Bartsch et al. 2019), we observed that the thus estimated parameters were significantly better than the parameters obtained directly by a Baum–Welch algorithm, most likely because of the applied missed event correction.

**Interplay** As we will demonstrate in Analysis of patchclamp recordings, model-free idealizations allow a standalone analysis of patchclamp recordings. Moreover, they can assist an HMM-based analysis in various forms (illustrated in Fig. 11): model-free idealization can help to identify and remove artifacts, we used for instance ${\text {JULES}}$ in (Bartsch et al. 2019) to assists an HMM-based analysis in that way. They can be used to determine the number of conductance levels (paragraph ’Analysis of the conductance levels’ in Analysis of patchclamp recordings) and help select and verify a specific Markov model (paragraph ’Selection and verification of a Markov model’ in Analysis of patchclamp recordings). Furthermore, most HMM-based parameter estimation approaches are iterative procedures which require starting values. Those are particularly crucial when the procedure converges to a local optimum only. Such starting values can be provided by previously obtained values using model-free idealizations. Finally, model-free idealizations and the resulting parameter estimates using a missed event correction can be used to verify HMM-based idealization and parameter estimates, and vice versa. This is particularly valuable as they have different strengths and weaknesses as outlined in the following paragraph.

We also note that the local deconvolution approach used in our model-free idealization methods, see (Pein et al. 2018), can be used to improve HMM-based idealizations, obtained, for instance, by a Viterbi algorithm, as our approach not only takes into account explicitly the filtering, but is also time-continuous. It only relies on a prior fit that fixes the number of conductance changes and their rough locations. It can be called by the function *deconvolveLocally* in the package *clampSeg*.

**HMM versus model-free idealization: compared and contrasted** In general, HMM-based approaches achieve a higher temporal resolution of gating dynamics because of their stronger assumptions. Hence, parameter estimates might be more accurate as they rely on more detected events. Moreover, HMMs allow for immediate parameter estimation and interpretation, which is often the main goal of an analysis. And since the HMM state space is fixed in advance, the idealization immediately assigns every time point to one of the states. In contrast, model-free idealizations often have to be postprocessed, (e.g., by clustering or thresholding) to identify discrete states, because conductance levels are determined freely.

On the other hand, there are several disadvantages, some of which are closely entangled with the advantages. As discussed above, the need for often extensive preprocessing adds subjectivity and also more potential sources of data analysis errors. In contrast, in such situations, model-free methods may right away provide a reasonable idealization as they can potentially handle inhomogeneity in a more flexible way, in particular those which act locally on the dataset. The state space and a model for the noise must be fixed in advance, thereby strongly limiting the possible results. Model selection always has a subjective component and can lead to a flawed idealization, for example by inadvertently modeling two states with similar but subtly different conductance or noise levels as only one state, or by prescribing an unsuitable noise model which can lead to detection of spurious state changes. Within an HMM framework, one can only incompletely determine whether the data are compatible with the underlying model assumptions. Hence, despite the above described advantages, at least in simulations and real data examples in Pein et al. (2021); Bartsch et al. (2019), we observed that parameter estimates based on an idealization (either obtained by model-free approaches or by the Viterbi algorithm) appear to be more accurate than direct estimates by the Baum–Welch algorithm. Gating dynamics are time-continuous processes, but for simplification, many HMM approaches underlie a time-discrete Markov chain as an approximation. A time-discrete approximation is also implied by most model-free approaches as they allow gating events only at the sampling points. An exception is the local deconvolution approach used in Pein et al. (2018, 2021).

Some of the subjectivity and other problems in HMM modeling can be mitigated by conducting a model-free idealization to inform preprocessing and model selection. In summary, HMM-based and model-free approaches can (and should) be used to complement each other to verify each other’s results. Artifacts and missed events might be reasons for some differences, but otherwise results should be similar.

### Review of existing model-free idealization approaches

Many analyses are still performed by visual inspection, often with manually chosen event times or in a semi-automatic way, for instance by amplitude thresholding (Colquhoun 1987; Sakmann and Neher 1995), as e.g., offered by pCLAMP 10 software (Molecular Devices), or by the semi-automatic *SCAN* software (Colquhoun and Sigworth 1995) which allows *time-course fitting*. Hence, those approaches are typically time-consuming and subjective. Moreover, approaches which are based on additional filtering (often by low-pass Gauss filters) aggravate detection of small events. A first approach for a fully automatic idealization was slope thresholding (Basseville and Benveniste 1983); for instance, ${\text {TRANSIT}}$ (VanDongen 1996). Recently, Gnanasambandam et al. (2017) proposed idealizations based on the minimal description length (${\text {MDL}}$). All of them (except the semi-automatic *SCAN* software) ignore low-pass filtering and hence may have difficulties to idealize events correctly on small temporal scales. Furthermore, if events are present on multiple scales (recall Figs. 1, 4, 6), uniscale thresholding procedures will usually fail.

**Fig. 12:**
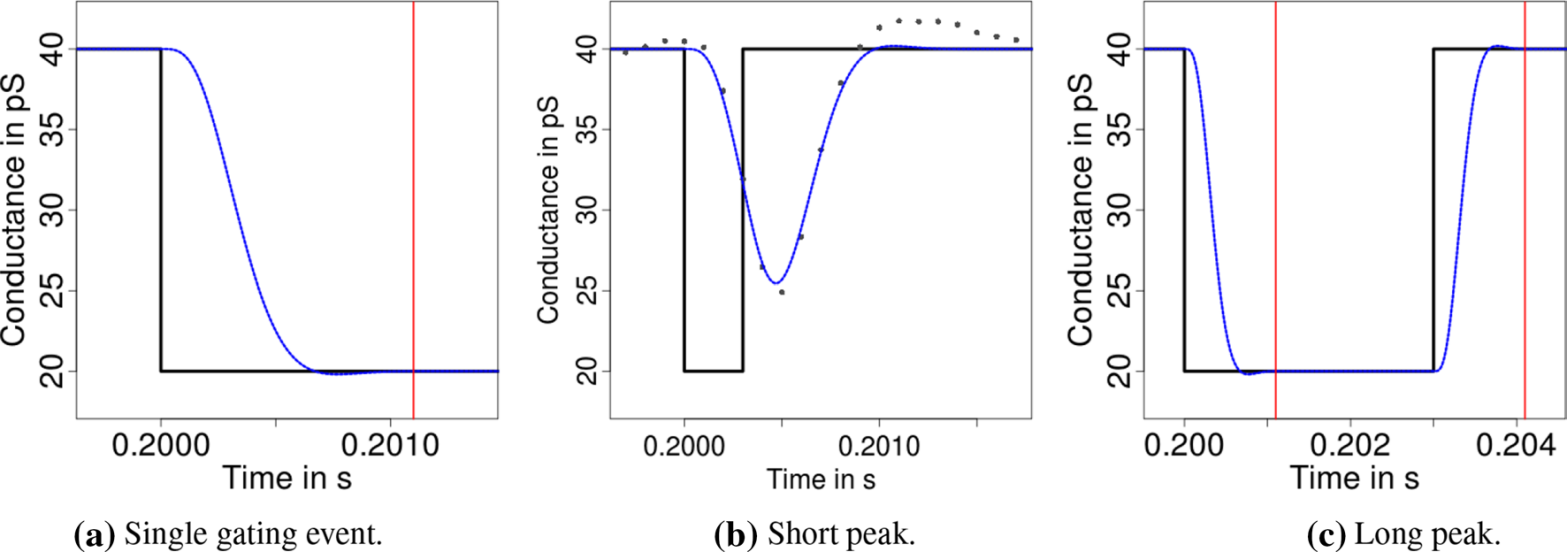
Signals (black line) containing a single gating event, a short peak, and a longer peak and their convolutions (blue line) with a four-pole low-pass Bessel filter with normalized cut-off frequency of 0.1 and sampling rate $10^4$. Vertical red lines indicate the event time plus the filter length $m / f_{\varvec{s}}$

As mentioned in the introduction, ${\text {JSMURF}}$ (Hotz et al. 2013), ${\text {JULES}}$ (Pein et al. 2018), and ${\text {HILDE}}$ (Pein et al. 2021) are multiscale procedures combined with local deconvolution and hence take into account both issues. Consequently, they provide usually more accurate results as demonstrated in simulations and real data applications. As described in the following, they mostly differ in how they take into account the filter when detecting events and hence whether they are suitable to detect short events, but also whether they incorporate the possibility to allow for heterogeneous noise. To understand the methodology better, it is illustrative to plot the convolution of a single gating event and single peaks with the kernel of a low-pass filter, see Fig. 12. We stress that for the short event displayed in Fig. 12b, the filtered signal does not reach the lower conductance level of the original signal. This is generally the case for peaks shorter than the filter length $m/f_{\varvec{s}}$. Hence, if such short events are present, deconvolution techniques are indispensable to idealize those conductance levels correctly.

**JSMURF** The **J**ump-**S**egmentation by **MU**lti**R**esolution **F**ilter, ${\text {JSMURF}}$, from Hotz et al. (2013), combines a multiscale criterion with rigorous error control to reliably detect events on various temporal scales simultaneously. More precisely, it takes into account all scales above the filter length, and for each of those intervals, it ignores the first *m* data-points. As illustrated in Fig. 12 only during these *m* point long transitions, the convolution is not matching the conductance *f*. It provides the following strict error control. The probability that the idealization contains at least one false-positive event (an event that is not contained in the true conductance *f*) is bounded by the error level $\alpha$. The original work (Hotz et al. 2013) assumed homogeneous noise, and (Pein et al. 2021) proposed an extension to heterogeneous noise.

**JULES** The **JU**mp **L**ocal d**E**convolution **S**egmentation filter, ${\text {JULES}}$, from Pein et al. (2018), applies a multiscale criterion to all temporal scales and combines it with a postfilter step to remove incremental steps as, for instance, occurring in Fig. 2. Finally, a local deconvolution approach is proposed to idealize short events well. The error level $\alpha$ bounds the probability of detecting a false positive approximately. All in all, ${\text {JULES}}$ is particularly designed for homogeneous noise and short events.

**HILDE** **H**eterogeneous **I**dealization by **L**ocal testing and **DE**convolution (Pein et al. 2021) obtains idealizations in three steps: It applies ${\text {JSMURF}}$ to detect events on large temporal scales; afterwards, it tests locally for additional short events. Those tests explicitly take filtering into account. The final idealization is once again obtained by local deconvolution. Local tests are performed on scales up to length $l_{\max }$. The error level $\alpha := \alpha _1 + \alpha _2$ is split between the multiscale criterion of ${\text {JSMURF}}$ (error level $\alpha _1$) and the local tests (error level $\alpha _2$). False positives occur again only with probability approximately $\alpha$.

**Simulation results** In the following, we give a brief qualitative summary about the simulation results in (Hotz et al. 2013; Pein 2017; Pein et al. 2018, 2021). Generally speaking, such computer simulations are a systematic but also computation intensive way to determine precisely how long an event has to be such that an idealization method is able to reliably detect it. However, we stress that all quantitative results depend on the signal-to-noise ratio, the filter, and on tuning parameters.

We found that ${\text {JSMURF}}$ reliably idealizes events of medium or large length (usually, an event has to be at least few times the filter length) even when the conductance change is small, confer (Hotz et al. 2013). This is essential to idealize *subgating* events. In comparison, ${\text {JULES}}$ and ${\text {HILDE}}$ are able to reliably idealize much shorter peaks, if they are isolated. Isolated means that two events have to be separated by at least three times the filter length if homogeneous noise is assumed but at least five times the filter length if heterogeneous noise is assumed (for a filter truncated after $m=11$ sampling points). Moreover, for a good idealization, events have to be usually only few sampling points long, but can be shorter than the filter length. Hence, those two approaches are suitable to idealize *flickering*. ${\text {HILDE}}$ allows events to be a bit shorter than ${\text {JULES}}$.

${\text {JSMURF}}$ is usually the fastest of our three approaches and an idealization of several hundred thousands up to a few million data points take often only seconds (when Monte Carlo simulations have already been performed). In comparison, an idealization of the same dataset with ${\text {JULES}}$ may last around a minute and with ${\text {HILDE}}$ few minutes. All run times are measured on a standard laptop and increase typically linearly in the number of data points. A notable exception are situations in which ${\text {JSMURF}}$ detects almost no change-points, and then, the run time increases quadratically in the number of observations. For instance, the idealization in Fig. 9 took roughly half an hour. Since ${\text {HILDE}}$ uses ${\text {JSMURF}}$ as a first step, its run time is similarly slow.

### Analysis of patchclamp recordings

In this section, we provide a step-by-step guide on how to analyze patchclamp recordings using model-free idealizations. In addition, we describe their interplay with Markov model-based analyses, see also the introduction and in particular Fig. 11 for an illustration. Of course, any analysis depends on the specific datasets and its goals. Hence, the following steps should be seen as more of general guidance that has to be interpreted flexibly. We also stress that it contains time-consuming verification steps which might not be necessary in every analysis.

**Fig. 13:**
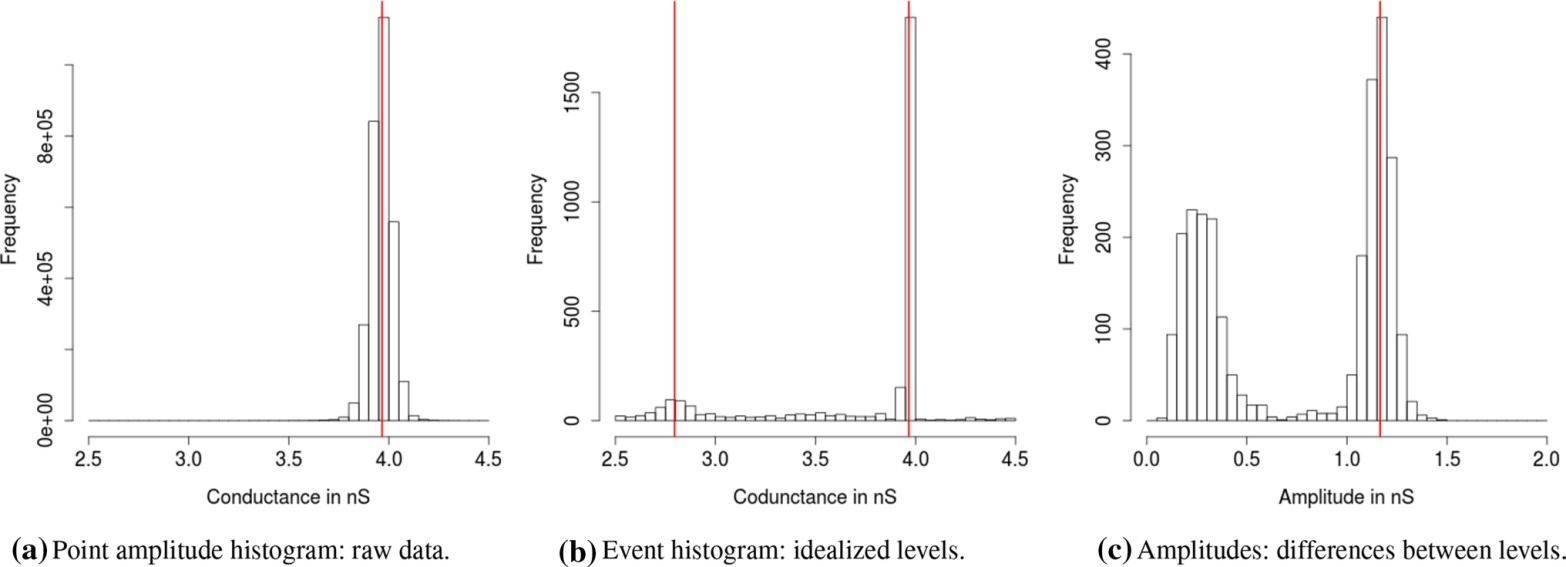
Histograms of the PorB measurement with ampicillin in Fig. 1. Histograms are based on the visualized and on ten additional traces. Code to obtain such histograms was explained in the paragraph ’Interpreting, plotting and verification of the output’ in Using our software. In the point amplitude histogram, we found one dominant conductance level of 3.9664 nS (estimated by the half sample mode). Smaller conductance levels are not visual, since they are too short and smoothed by the low-pass filter (in total, 2,476 data points are between 2.5 nS and 3.5 nS). The event histogram confirms this conductance level. Note that the peak is much narrower in the event than in the point amplitude histogram. This is usually the case and improves identification of conductance levels. Moreover, because of the deconvolution step in our idealization and since dwell times are not represented in the event histogram, we were also able to identify a second conductance level of 2.7956 nS, i.e., the amplitude (difference, blockage effect of the ampicillin) is 1.1708 nS. The amplitude histogram confirms this finding with a pronounced mode at 1.1662 nS. A simple mean is roughly the same with 1.1546 nS. The amplitude histogram but also the event histogram shows further events. Those events could be matched to processes unrelated to the interaction of ProB and ampicillin, and hence should be ignored for the ampicillin influence, confer (Bartsch et al. 2019)

**Analysis of the conductance levels** For this step, we assume that the underlying protein attains only a finite number of conformations and hence that only a finite number of conductance levels occur. We aim to determine this number, the values of the conductance levels, and possible transitions between those levels. This can be done in various ways and we will only sketch important ideas. Event histograms (histograms of the idealized conductance levels) and amplitude histograms (histogram of the differences between consecutive segments in the idealization) should be used as a visualization of the underlying conductance levels, see Fig. 13.

The idealized conductance levels form a mixture distribution, typically a Gaussian mixture, around the true conductance levels, where randomness results from measurement and idealization errors (and hence the peaks are narrower if those are better performed). Modes correspond to the true conductance levels. An example can be found in Fig. 13. One can use simple approaches based on a Gaussian assumption to estimate modes. We obtained good results using the half sample mode (Robertson and Cryer 1974), because it is quite robust against outliers. In more difficult cases, where peaks cannot be identified that clearly, more involved statistical methodology to estimate the components of a mixture distribution has to be used; for an overview, see (McLachlan and Peel 2004) and the references therein, or the accuracy of the measurement or idealization has to be increased.

Subsequently, one often aims map idealized conductance levels to their corresponding mixture components. This can, for instance, be done by defining non-overlapping intervals around each estimated conductance level (mixture component) and assigning all events whose estimated idealized conductance level lies within an interval to the corresponding mixture component’s conductance level. It is often a good idea to remove segments that are far from any estimated conductance level from the subsequent analysis, i.e., assigning such idealized conductance level to no interval, since they typically result from artifacts. Note that idealization methods are often sensitive enough to detect baseline fluctuations and fluctuations due to pink noise as events. As a result, often several consecutive events are within the same interval and should be interpreted as one segment only. In other words, this process can also merge segments and thus remove spurious events.

**Selection and verification of a Markov model** As discussed before, a time-continuous Markov model is a common assumption to analyze patchclamp recordings. Since model-free idealizations are obtained without any prior assumption on the gating dynamics, they can be used to determine and verify a Markov model. To avoid statistical dependency, a careful analysis involves splitting the measurements and using the first part to select a Markov model and the second part to verify the model. Recall that a Markov model has two key properties: dwell times (how long a channel stays in one Markov state) are independent of each other and are exponentially distributed. Since it is often simpler, one might aim to verify uncorrelated, instead of independent, dwell times, though lack of correlation does not imply independence. When checking whether dwell times follow a Markov model, one has to take into account that short events might be missed. Nonetheless, at least in simple Markov models with only few states, one readily can check for uncorrelated and exponentially distributed dwell times; for an example, see (Bartsch et al. 2019, Fig. S4 in the supplement).

**Parameter estimation** Once a specific Markov model is assumed, one has to estimate its parameters. To this end, it is essential to take into account missed events. Missing events shorter than a certain resolution limit are widely discussed in the literature. The exact distribution is calculated by Hawkes et al. (1990), an estimator called ${\text {MIL}}$ of the Q-matrix is suggested by Qin et al. (1996) and integrated in the ${\text {QuB}}$ software package (Nicolai and Sachs 2013), the exact maximum-likelihood estimator for the Q-matrix for two conductance levels is obtained by Colquhoun et al. (1996), and recently, a Bayesian approach was proposed by Epstein et al. (2016). In Pein et al. (2018, (2021); Bartsch et al. (2019, (2020), we applied simpler approximations, which worked well, since the measurements could be modeled well by Markov models with only two or three states.

**Verification using hidden Markov approaches **This step was already discussed in Hidden Markov Models. The previous analysis using model-free idealizations can be an essential help to perform an analysis using HMM-based approaches. HMM-based approaches are, however, potentially able to achieve better temporal resolution. Hence, both approaches should be used to verify each other’s results, both in terms of parameter estimation and of idealizations.

## Discussion

We gave detailed guidance on how to obtain model-free idealizations using ${\text {JSMURF}}$, ${\text {JULES}}$, and ${\text {HILDE}}$, and on how to use those idealizations together with HMM-based approaches to analyze patchclamp recordings. We believe that this provides a rather comprehensive toolkit for the analysis of many patchclamp recordings.

A notable exception are experiments with varying conductance. Such experiments are interesting, since not only the present value of voltage affects the channel, some channels are also affected by the present rate of voltage change. This includes channels that show no gating when the voltage is constant, but can be activated by a varying voltage. For other channels, different dynamics are observed when the voltage changes. One example is the protein channel Tim23 which tends to close when larger voltage levels are applied constantly (Denkert et al. 2017). Moreover, experiments with a constant voltage only allow to examine the gating dynamics at few voltage levels (or require large experimental effort), while with varying voltage, the dynamics can be analyzed for a whole range of voltages by a single experiment. Brief ideas were discussed in (Pein 2017, Sect. 6. Using our software) and (Diehn et al. 2019).

Though model-free approaches are in general more robust to artifacts than HMM-based approaches, confer (Pein et al. 2018, 2021) who demonstrated for ${\text {JULES}}$ and ${\text {HILDE}}$ certain robustness to model violations, there is need for improved methodology (either model-free or HMM-based) with a larger focus on robustness.

## Supplementary Information

Below is the link to the electronic supplementary material.

Supplementary file1 (pdf 1553 KB)

Supplementary file2 (zip 64 KB)

## Footnotes

1 All data were measured at the Steinem lab, Institute of Organic and Biomolecular Chemistry, Georg-August-University of Göttingen.

2 Strictly speaking the terminology ’model-free’ is misleading as also model-free approaches require an underlying model to be valid. However, we use this terminology mainly for historical reasons. The precise (non-parametric) models underlying our methodology are reviewed in  “Models”. We only assume that the underlying conductance is piecewise constant, but make no further assumptions about the gating dynamics.

3 https://www.r-project.org/.

4 https://cran.r-project.org/.

5 https://github.com/FlorianPein/clampSegGUI.

6 http://www.stochastik.math.uni-goettingen.de/dbacf.

7 Strictly speaking, this models only *heteroscedasticity*, one special form of heterogeneous noise that is, for instance, caused by open-channel noise. However, we expect our methods also to be robust to other forms of heterogeneous noise, confer the simulation results in Pein et al. (2021).
