# Supplementary material for: Analysis of patchclamp recordings: model-free multiscale methods and software
Source: Eur Biophys J. 2021 Apr 9;50(2):187–209. doi: 10.1007/s00249-021-01506-8 (PMC8071803; doi:10.1007/s00249-021-01506-8)
Supplement: Supplementary file 1 — Supplementary file1 (pdf 1553 KB) [file 249_2021_1506_MOESM1_ESM.pdf]

Supplement to
Analysis of Patchclamp Recordings: Model-Free Multiscale Methods and Software

Florian Pein, Benjamin Eltzner, Mariyam Khan, Florian Ebmeier,
Stanislav Syekirin and Axel Munk

In the following we describe how to technically operate the graphical user interface (GUI) **clampSegGUI** as referenced to in Section II-A in the main paper. The GUI is available on Github. Please see `installation.pdf` for an installation guide.

Start

From the top level directory of the program, where `run.pyw` is, run the program:

```
python run.pyw
```

Here, `python` should refer to at least Python 3.5. If there are multiple installations of Python on your system, your command might need to be more specific, e.g. `python3 run.pyw` or `python3.5 run.pyw` .

You should see something like this:

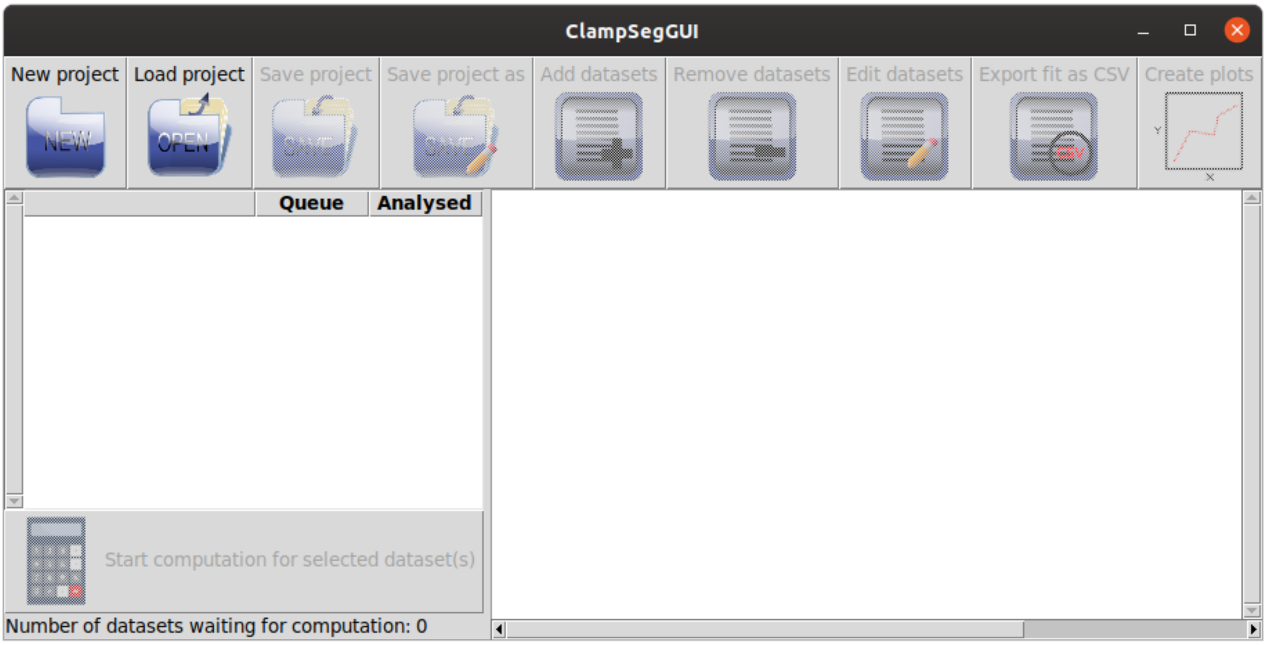

Projects

To start a new project, click on the `New project` button. To load a previous project click on the `Load project` button.

Assuming you started a new project, it will look something like this:

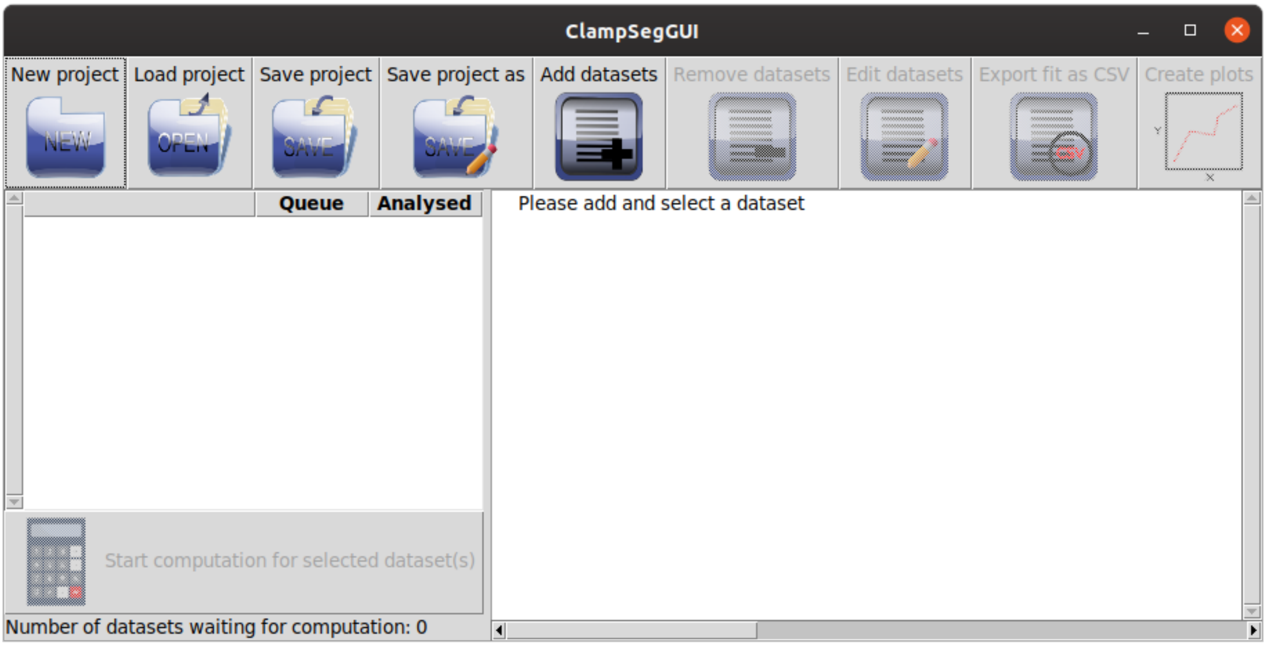

A click on the `Load project` button will start a filedialog asking you to select the project. The project should have the ending *.csg*. Note that this filedialog, and most other filedialogs, will freeze the main window for as long as the filedialog is open.

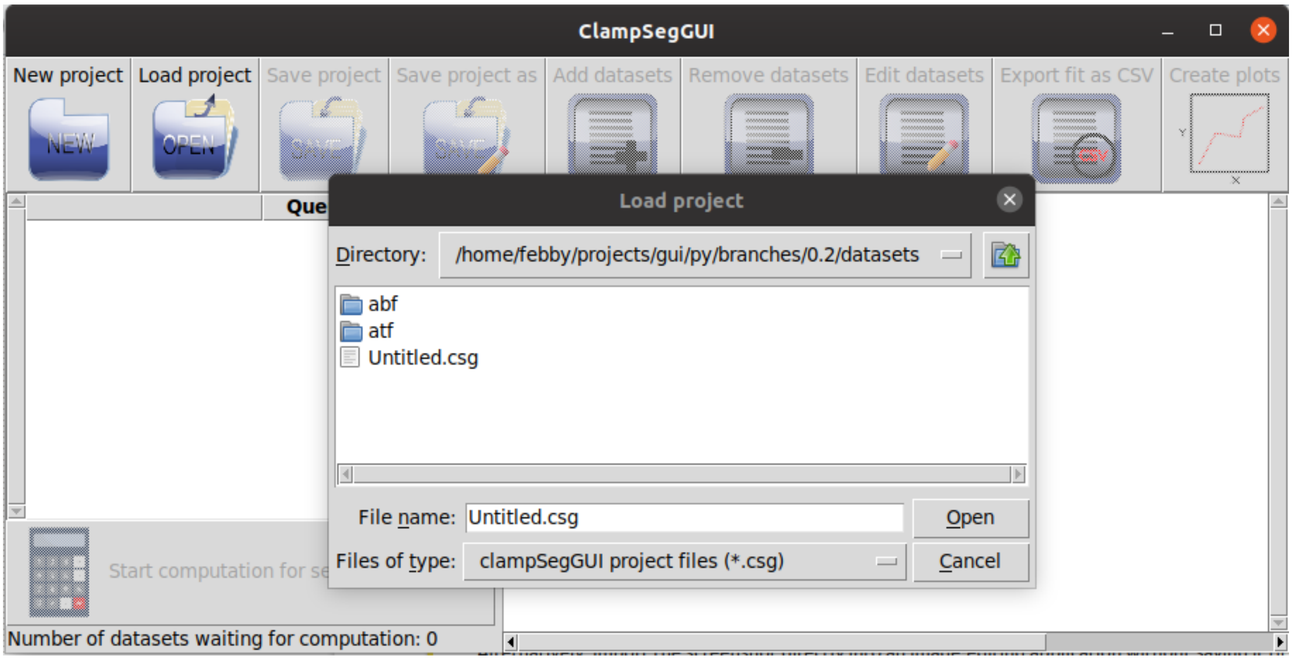

To save your project press the `Save project` or the `Save project as` button. If this project was a new project or you pressed the `Save project as` button, a filedialog will open, asking you to provide a filename.

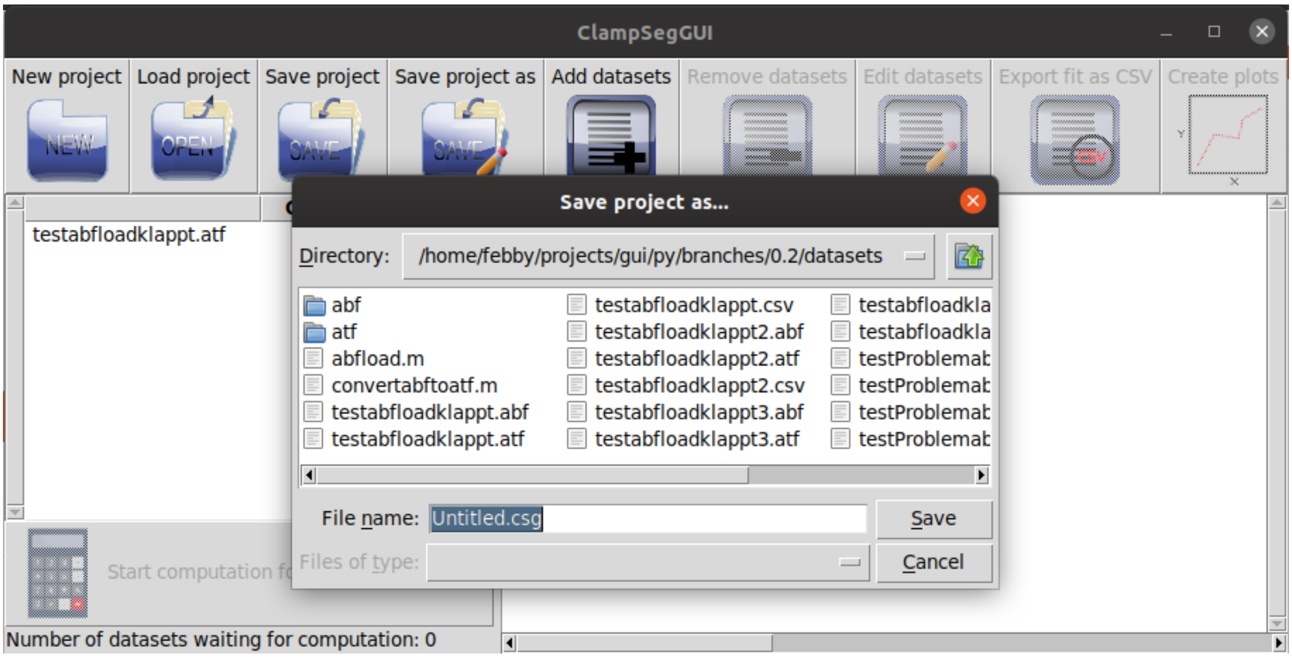

Datasets

Adding and removing datasets

To add one or multiple datasets, click on the `Add datasets` button. This will open a filedialog:

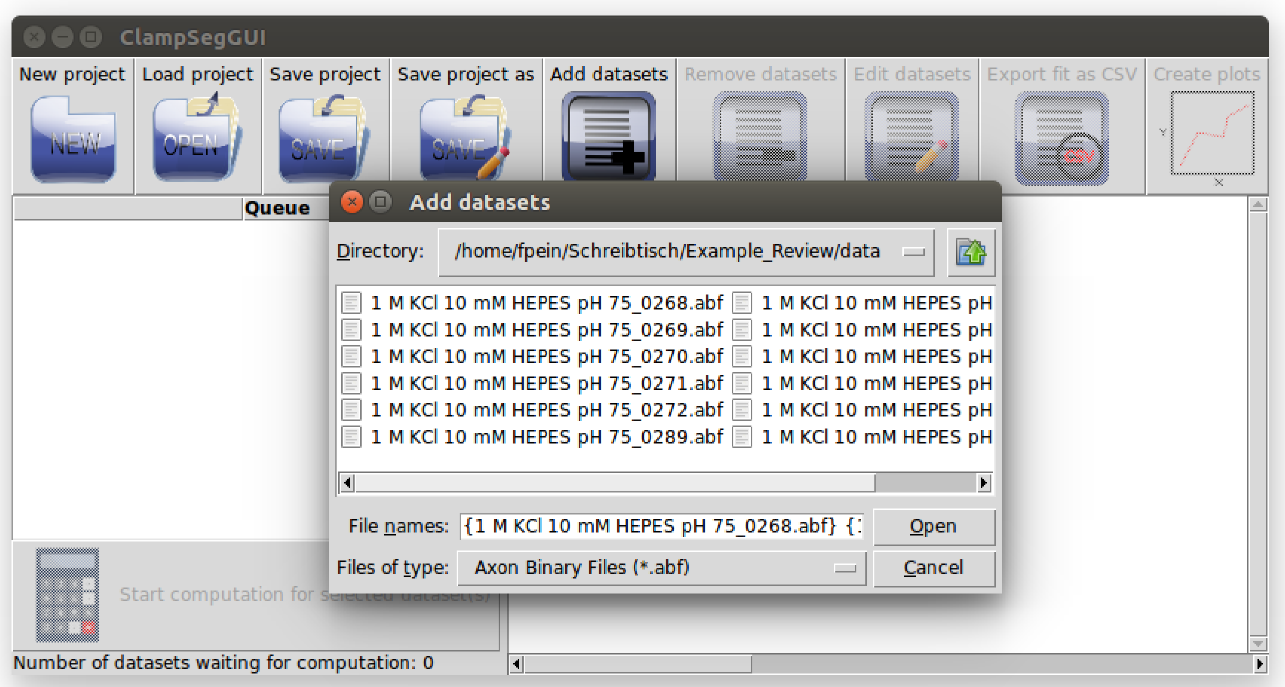

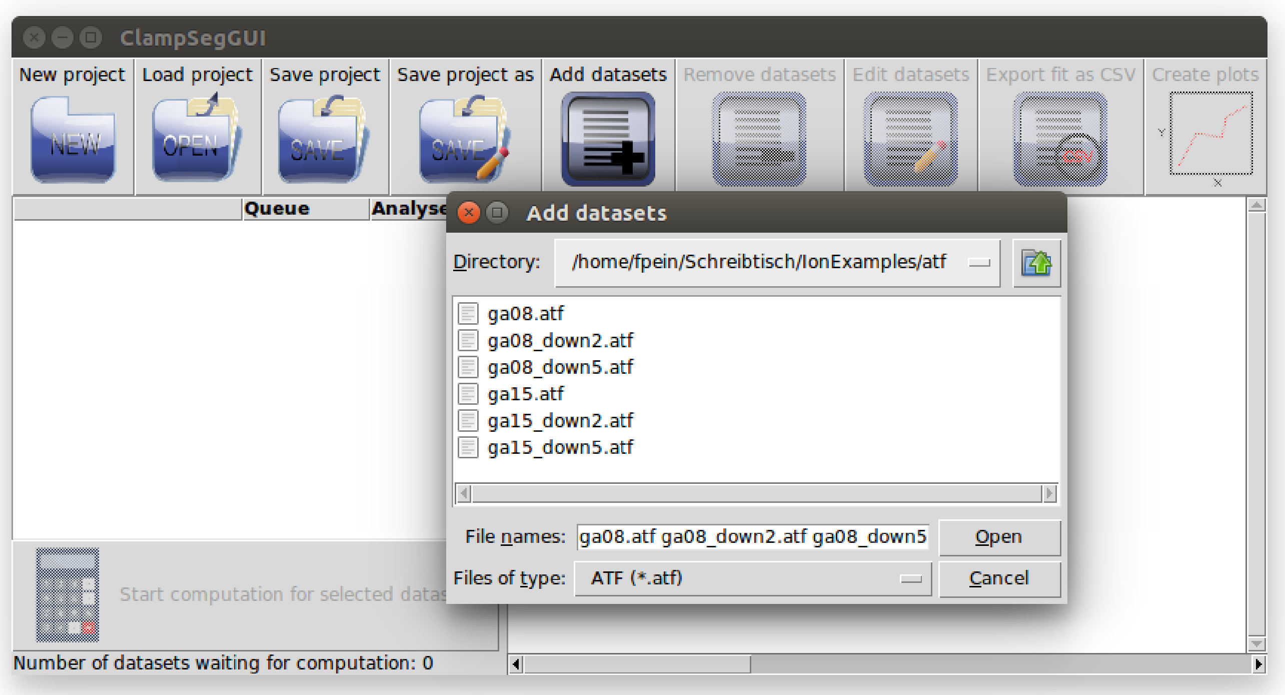

Here you can select one or multiple datasets, with filename extension *.abf* or *.atf*. Reading a file with a mislabeled ending will result in an error.

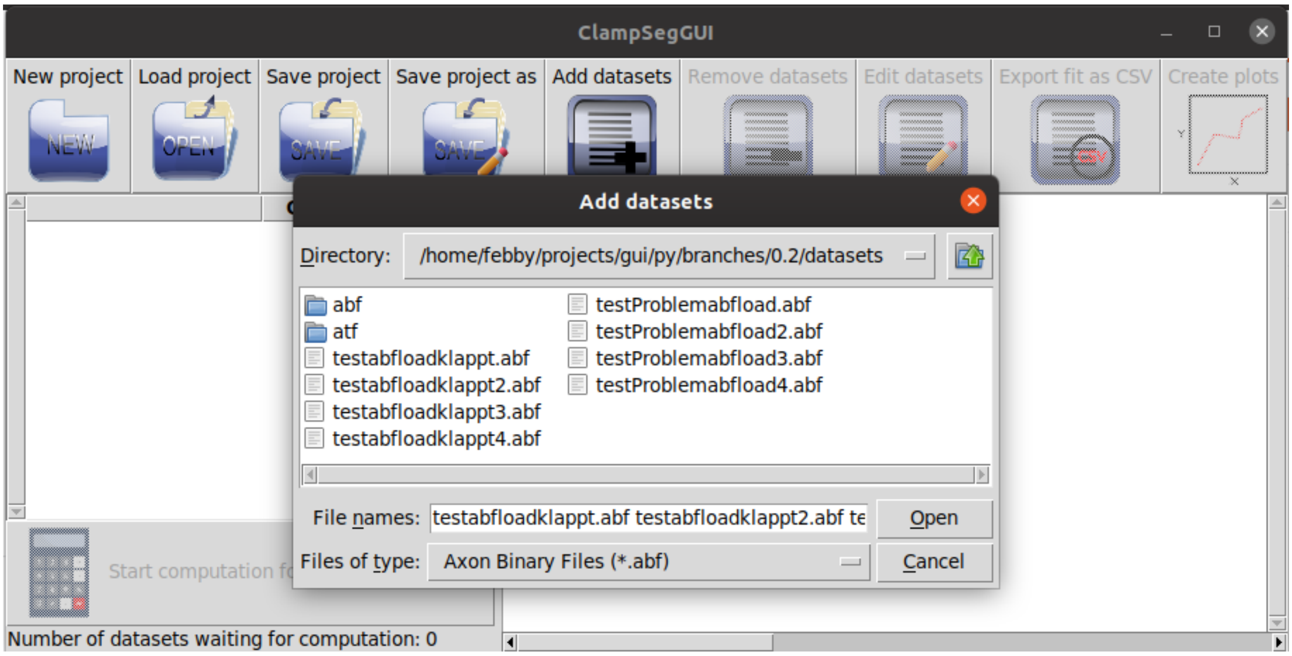

Clicking on the `Open` button will, after a short wait, open a window asking you to select a channel, optionally divide it by another channel and ask you for the appropriate unit of the result.

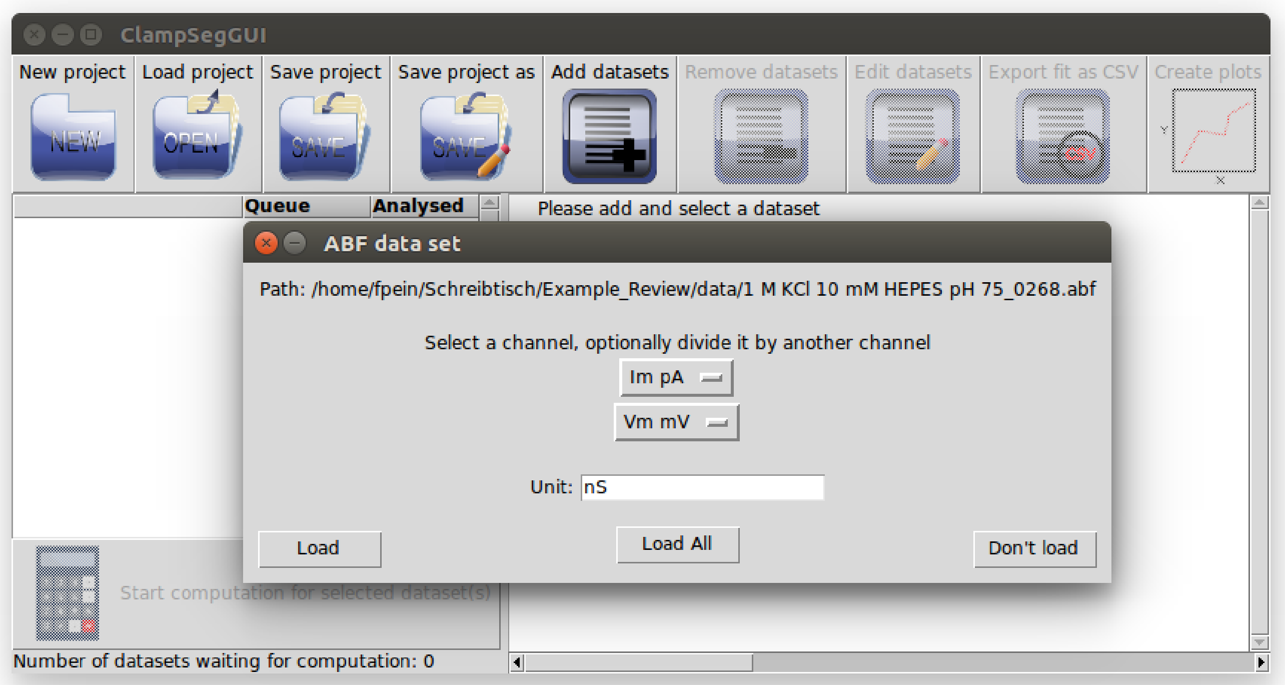

There are two options available to load datasets. Clicking on `Load All` will load all datasets, which have the same number of channels, same channel names and same channel units as the first dataset in the queue. If there are datasets in queue with different parameters, the window will pop up again. Alternatively, clicking on `Load` will only load the first dataset in queue and the window will pop up again (if there are further datasets to be loaded), which allows customizing loading for each dataset individually.

To remove datasets from the project, select all datasets you want to remove on the left. Then click on the `Remove dataset` button. A window will open and ask you to confirm. Click `Ok` to remove the selected datasets from the project.

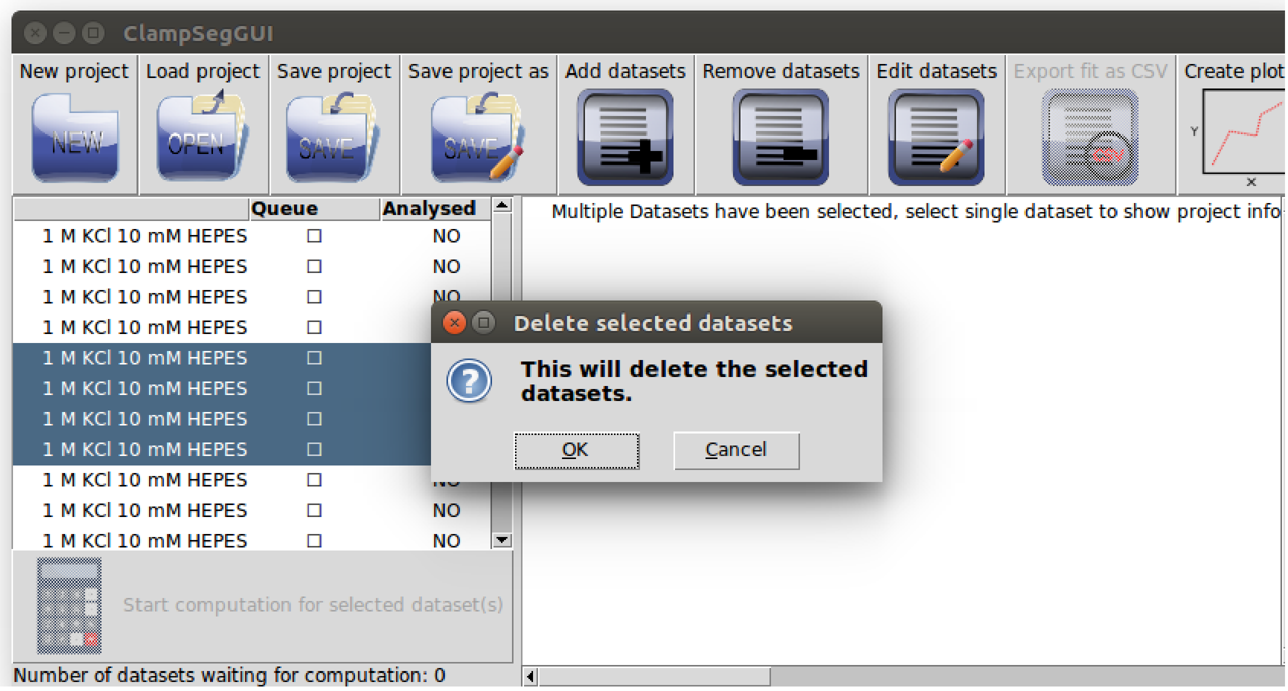

Viewing and editing metadata

To view the metadata of a dataset, select a single dataset on the left and the metadata will be shown on the right side.

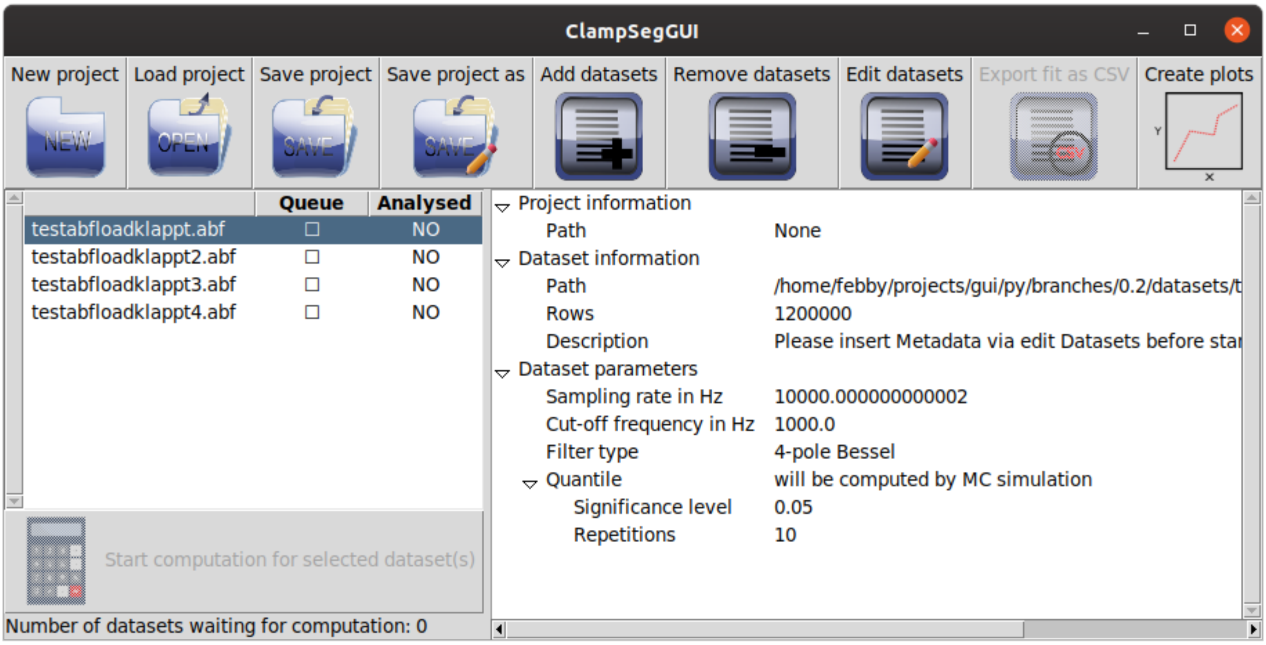

To edit the metadata of a dataset select all datasets for which you want to change metadata and press the `Edit datasets` button. This will open a new window, where you can input the metadata. This includes a description of the filter, the desired method to calculate a fit and its tuning parameters (details are explained below). Clicking on `Apply` will set all metadata that were changed for all selected datasets.

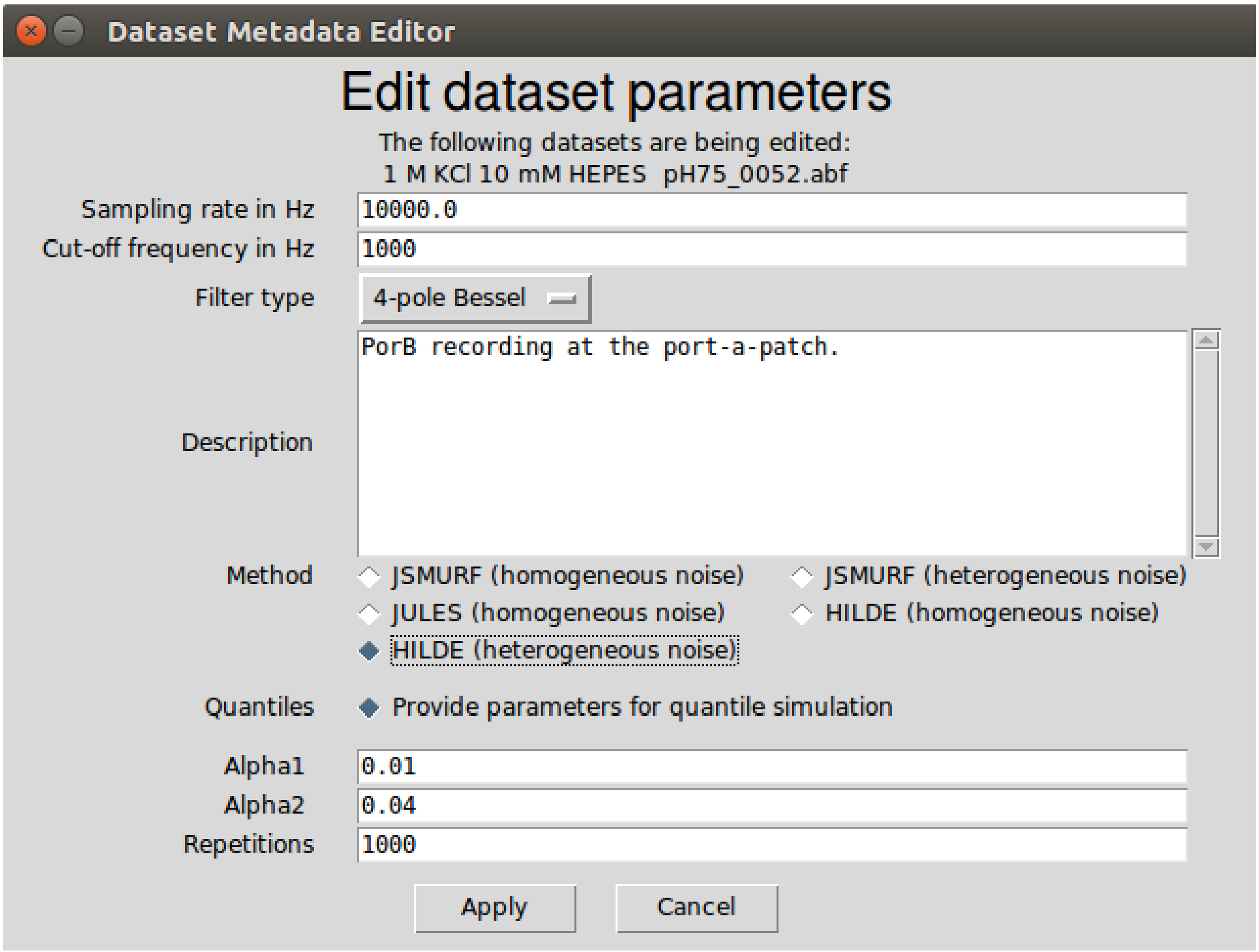

Filter parameters

Our methods require a correct specification of the used lowpass filter. So far only Bessel filters are supported. The following parameters can be specified.

- Sampling rate in Hz

  The sampling rate of the dataset. This is read from the input file, but should be checked.

- Cut-off frequency in Hz

  The cut-off frequency of the lowpass filter. Default value is `NA` and it must be configured before an idealization can be computed for this dataset.

- Filter type

  The type of the lowpass filter, currently only Bessel filters with different number of poles are supported. Default value is `NA` and it must be configured before an idealization can be computed for this dataset.

Description

An option to describe the data set for the users convenience.

Method

Radio buttons for selecting the idealization method. All three approaches JSMURF, JULES and HILDE can assume homogeneous noise, but only JSMURF and HILDE allow for heterogeneous noise. Hence, there are five methods to choose from: `JSMURF (homogeneous noise)` , `JSMURF (heterogeneous noise)` , `JULES (homogeneous noise)` , `HILDE (homogeneous noise)` and `HILDE (heterogeneous noise)` . See Section II-B in the main paper to decide which method is suitable for a given dataset.

Tuning parameters

Which tuning parameters can be chosen depends on the selected method. All parameters can be tuned to achieve better idealizations under specific circumstances. We advise to leave the default parameters unchanged unless specific reasons exist. Further details are given in the main paper.

Similar to the *R* code, quantiles (critical values) are required to distinguish between events and noise. The quantiles are usually computed by Monte-Carlo simulations. Monte-Carlo simulations may take a while, depending on the size of the dataset and the number of repetitions it may even last up to a few hours. They are stored and loaded automatically in the background. Only at the first time (unless the R package `R.cache` was used before) the user is asked to confirm that a folder is created to store those computations permanently on the disk (they can be deleted at any time). If the user declines it, a temporary folder is used and Monte-Carlo simulations have to be computed the next time again.

- Provide parameters for quantile simulation / Provide quantile

`JSMURF (homogeneous noise)` and `JULES (homogeneous noise)` rely on a single quantile, which can be preassigned by the user (e.g. based on previous calculations) as an alternative to Monte-Carlo simulations. We strongly recommend to the inexperienced user to perform Monte-Carlo simulations. Select `Provide parameters for quantile simulation` if you want to perform MC simulations and `Provide quantile` if you want to provide the quantile directly. This changes which of the lower parameters can be selected.

- Quantile

  A real-valued number giving the quantile.

- Alpha

  A probability (a real-valued number between 0 and 1) giving the error level α.

- Alpha1, Alpha2

  For HILDE the significance level α is split between `Alpha1` α_1 and `Alpha2` α_2 ($\alpha = \alpha_1 + \alpha_2$).

- Repetitions

  An integer giving the number of repetitions r for the Monte-Carlo simulations. Repetitions are individually selected and stored for the two HILDE methods and for JSMURF and JULES.

Calculating and exporting idealizations

Before the calculation for a dataset is possible, the cut-off frequency and the filter type have to be specified via `Edit datasets` .

To put datasets into the queue for calculating fits or removing them from the queue click on the empty / filled checkbox on the left in the column `Queue` (marked in red in the following picture). A new calculation overwrites the old idealization. To obtain two idealizations for the same dataset, it is required to add the dataset a second time to the project.

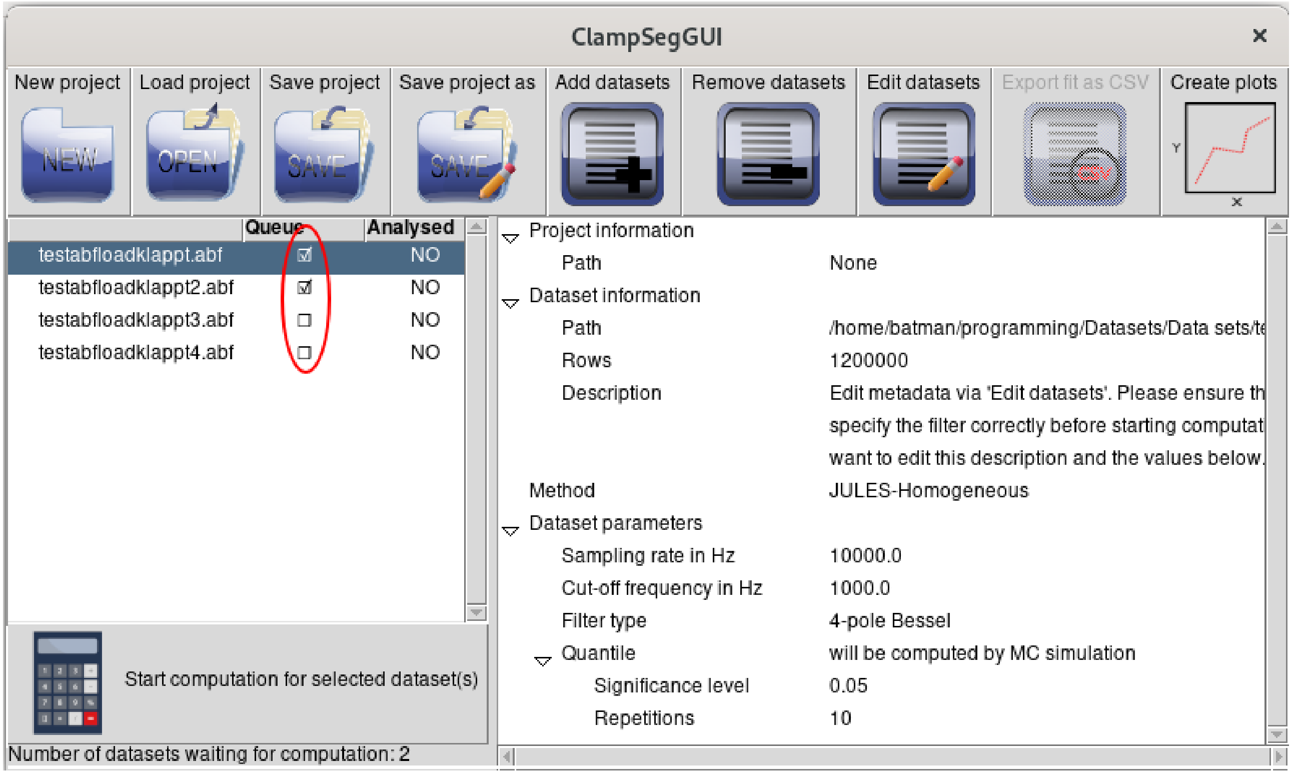

If the queue is not empty, click on the button marked `Start computation for selected dataset(s)` to start the computation. This will open a new window informing you that the computation is currently running. Note that this may take a while, depending on the the size of the dataset and the number of repetitions for the Monte-Carlo simulations. All datasets in the queue are computed step by step. For each one the computation starts with Monte-Carlo simulations if required and then with the main calculation.

You can stop the calculation of the quantile by clicking on the `Stop!` button of the window.

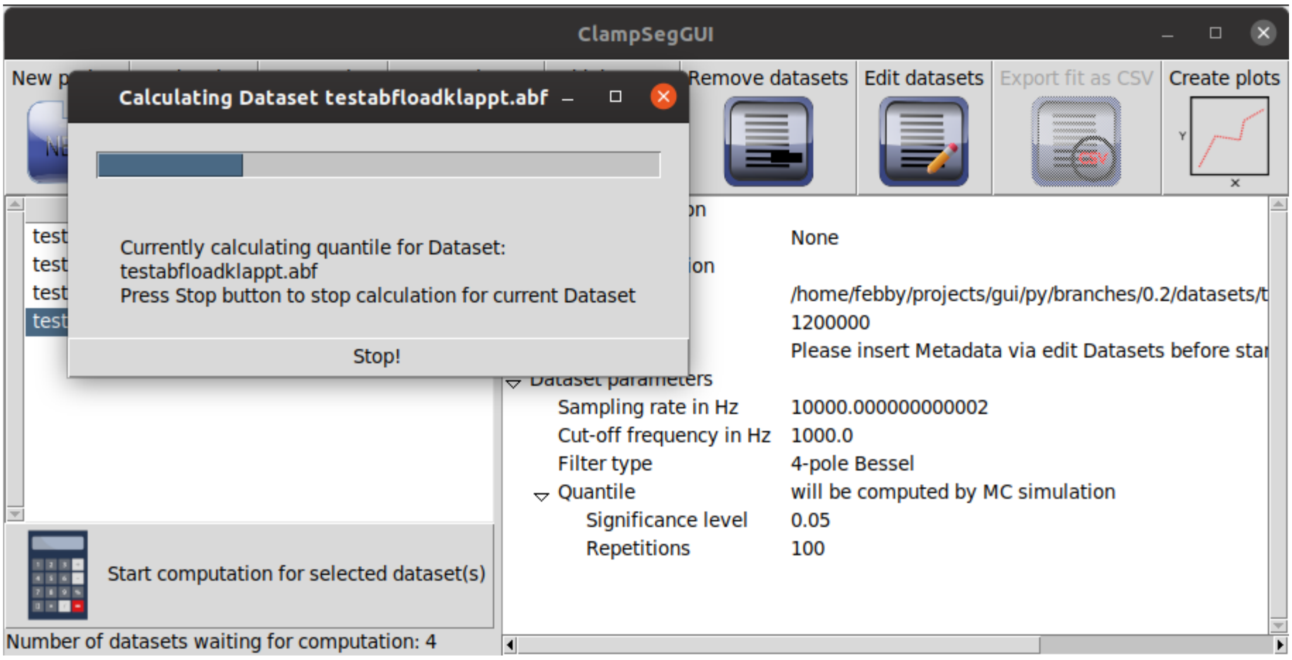

The calculation of the idealizations can currently not be stopped via a button and might take several minutes.

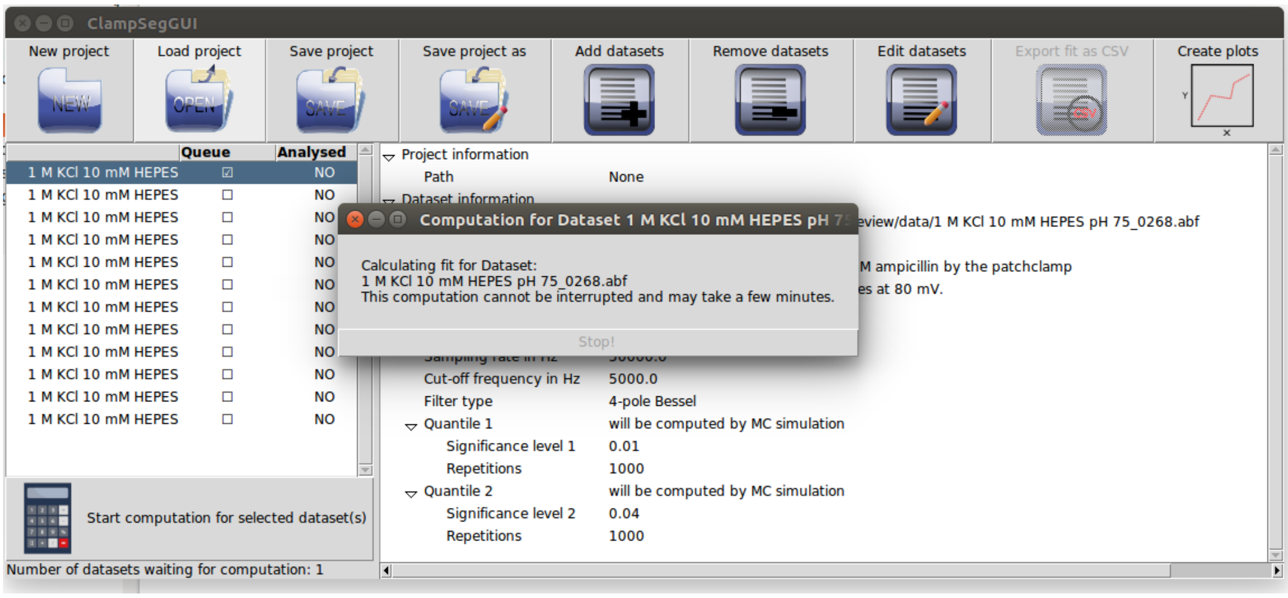

Once the idealizations are calculated, you can export the idealization as *csv* files. To do so, click on the `Export fit as CSV` button. This will open a filedialog for every selected dataset, asking you to specify a filename.

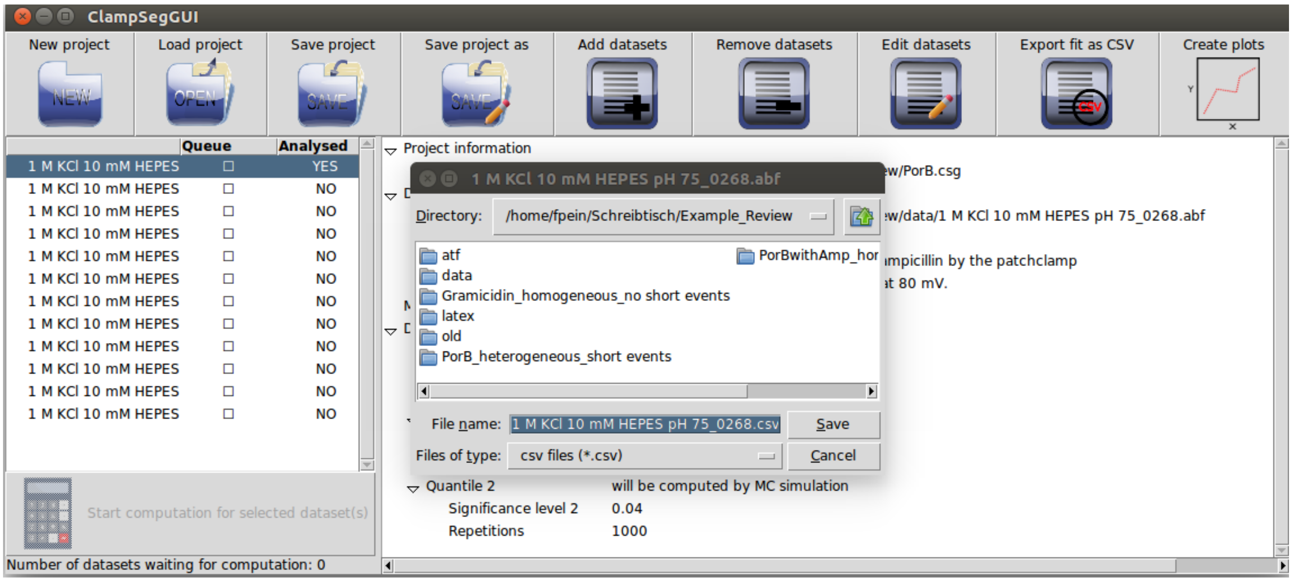

The resulting *csv* file will have a header as the first line. The data afterwards is comma separated. The file can be opened with any texteditor or spreadsheet program.

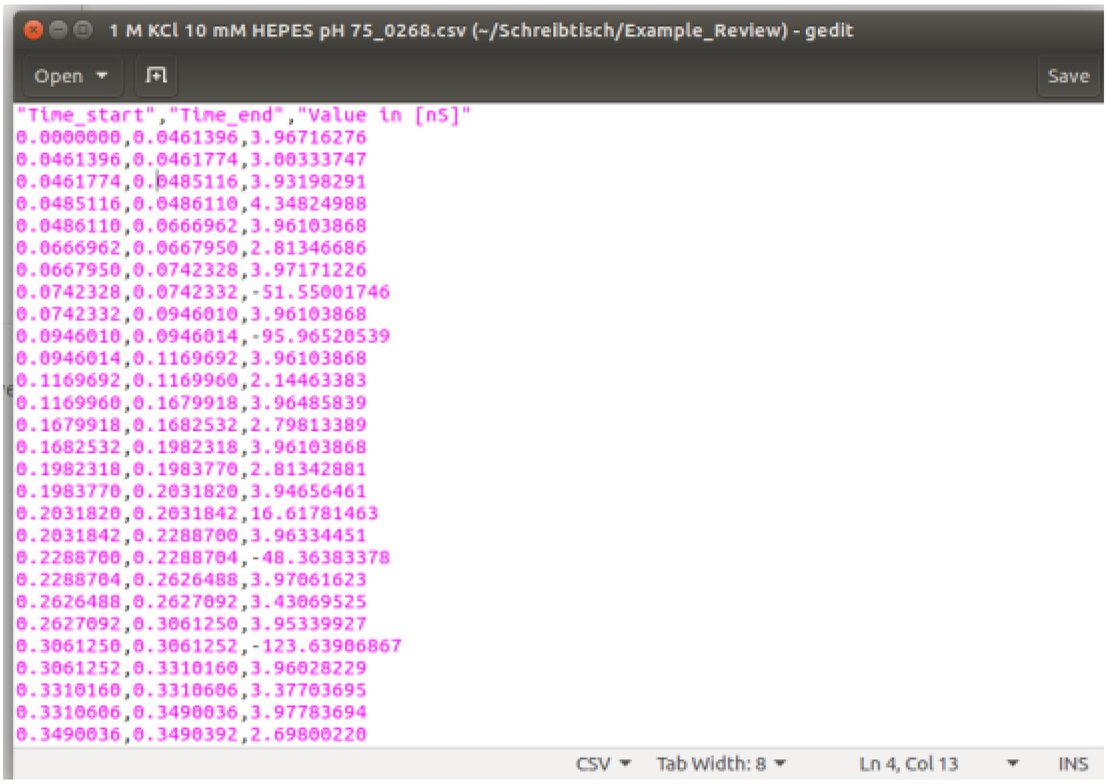

Creating plots

To create a plot of the data, select one or multiple datasets and press the `Create plots` button. This will open a new window for every dataset selected. In this window you can click the `Plot histogram` button to create a histogram plot of the data. If the fit for the dataset has been calculated, it will appear in the plot as a red line.

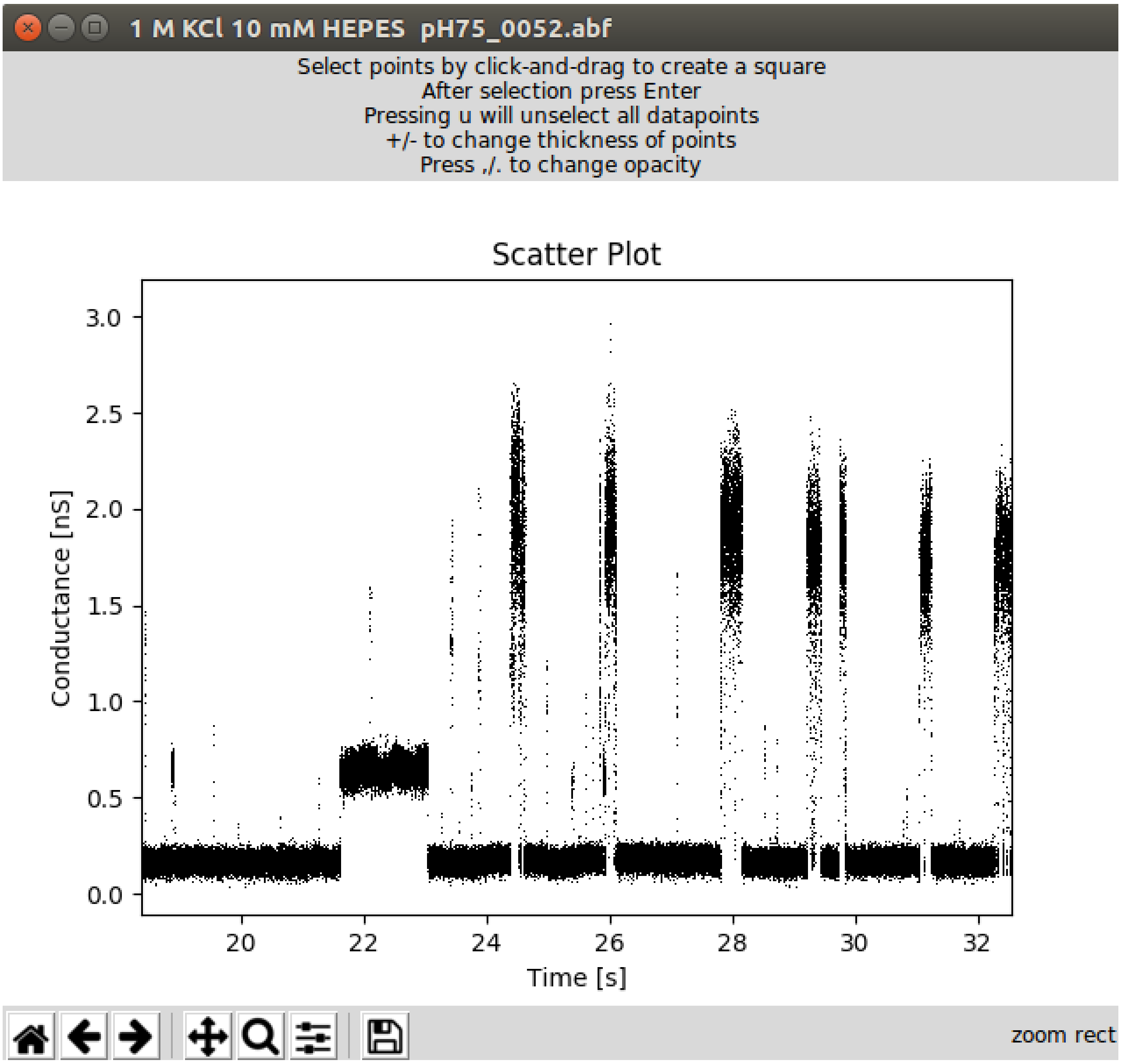

The plot can be saved by clicking on the save icon.

Reporting issues

Please send bug reports or any other feedback to the developers by emailing Florian Pein. Your feedback is highly appreciated.

Acknowledgements

The support of Deutsche Forschungsgemeinschaft grant SFB 803 Z2 is gratefully acknowledged.

License

```
This program is free software: you can redistribute it and/or modify
it under the terms of the GNU General Public License as published by
the Free Software Foundation, either version 3 of the License, or
(at your option) any later version.
```

This program is distributed in the hope that it will be useful,
but WITHOUT ANY WARRANTY; without even the implied warranty of
MERCHANTABILITY or FITNESS FOR A PARTICULAR PURPOSE. See the
GNU General Public License for more details.

You should have received a copy of the GNU General Public License
along with this program. If not, see <https://www.gnu.org/licenses/>.
