# Supplementary figures and images for: Analysis of patchclamp recordings: model-free multiscale methods and software
Source: Eur Biophys J. 2021 Apr 9;50(2):187–209. doi: 10.1007/s00249-021-01506-8 (PMC8071803; doi:10.1007/s00249-021-01506-8)

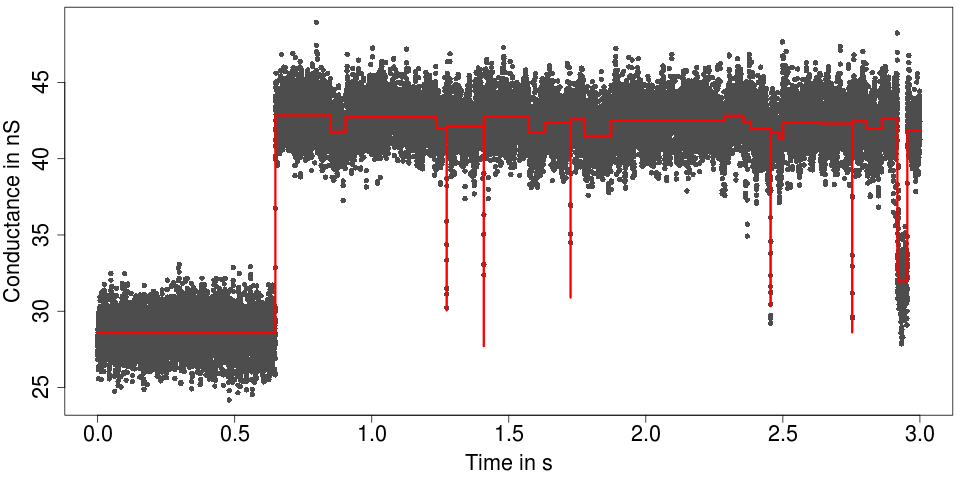

Supplement: Supplementary file 2 — Supplementary file2 (zip 64 KB) [file 249_2021_1506_MOESM2_ESM.zip › tutorial/comparePlotFull.png]

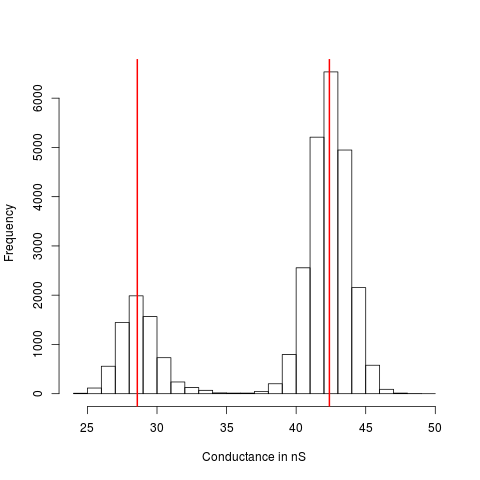

Supplement: Supplementary file 2 — Supplementary file2 (zip 64 KB) [file 249_2021_1506_MOESM2_ESM.zip › tutorial/compareHistogram.png]

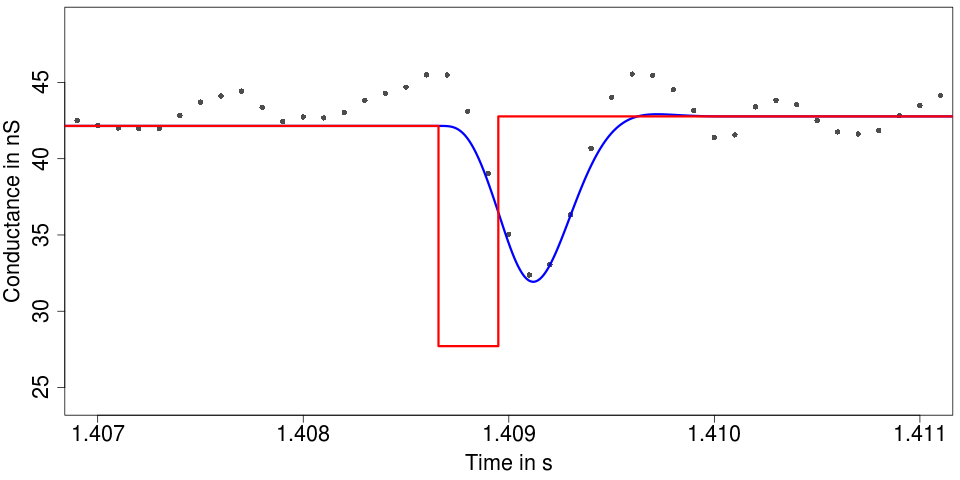

Supplement: Supplementary file 2 — Supplementary file2 (zip 64 KB) [file 249_2021_1506_MOESM2_ESM.zip › tutorial/comparePlotZoom.png]
